# Phylogenomic analysis of MKKs and MAPKs from 16 legumes and detection of interacting pairs in chickpea divulge MAPK signalling modules

**DOI:** 10.1038/s41598-017-04913-0

**Published:** 2017-07-10

**Authors:** Savithri Purayannur, Kamal Kumar, Vemula Chandra Kaladhar, Praveen Kumar Verma

**Affiliations:** 1 Plant Immunity Laboratory, National Institute of Plant Genome Research, Aruna Asaf Ali Marg, New Delhi, 110067 India; 2 School of Life Sciences, Central University of Gujarat, Gandhinagar, 382030 Gujarat India

## Abstract

The mitogen-activated protein kinase (MAPK)-mediated phosphorylation cascade is a vital component of plant cellular signalling. Despite this, MAPK signalling cascade is less characterized in crop legumes. To fill this void, we present here a comprehensive phylogeny of MAPK kinases (MKKs) and MAPKs identified from 16 legume species belonging to genistoid (*Lupinus angustifolius*), dalbergioid (*Arachis* spp.), phaseoloid (*Glycine max*, *Cajanus cajan*, *Phaseolus vulgaris*, and *Vigna* spp.), and galegoid (*Cicer arietinum*, *Lotus japonicus*, *Medicago truncatula*, *Pisum sativum*, *Trifolium* spp., and *Vicia faba*) clades. Using the genes of the diploid crop chickpea (*C*. *arietinum*), an exhaustive interaction analysis was performed between MKKs and MAPKs by split-ubiquitin based yeast two-hybrid (Y2H). Twenty seven interactions of varying strengths were identified between chickpea MKKs and MAPKs. These interactions were verified *in planta* by bimolecular fluorescence complementation (BiFC). As a first report in plants, four intra-molecular interactions of weak strength were identified within chickpea MKKs. Additionally; two TEOSINTE-BRANCHED1/CYCLOIDEA/PCF (TCP) transcription factors of class I were identified as novel down-stream interacting partners of seven MAPKs. We propose that this highly reliable MAPK interaction network, presented here for chickpea, can be utilized as a reference for legumes and thus will help in deciphering their role in legume-specific events.

## Introduction

For the entire duration of their life-cycle, plants are exposed to biotic and abiotic stresses. It is imperative for plants to sense the changes in their surroundings and relay the message to control the transcription of genes or activate the pre-existing proteins that help them cope with the change. Thus, signalling molecules are important components of plant systems for growth, development, and defence. The mitogen-activated protein kinase (MAPK) cascades are conserved signalling modules in eukaryotes. They sense extracellular and developmental cues and translate them into intracellular signals. In general, this three-tiered linear phosphorylation cascade involves the MAPK kinase kinase (MAP3K/MAPKKKs/MEKKs), the MAPK kinase (MAP2K/MKK/MEK) and the MAPK. However, exceptions in this linear cascade are known^1, 2^. These three proteins follow a sequential phophorylation mechanism to relay signals perceived by upstream sensors/receptors. The MAPK cascade has been characterized for varied plant processes including hormone signalling, development and differentiation, leaf senescence, floral abscission, abiotic stresses and defence response^3–6^.

Members of the MAPK modules have been identified from various plant species. *Arabidopsis thaliana* has 10 MKKs and 20 MAPKs^7^. Various attempts have been put forward to understand the intricacies of the MAPK interaction networks. MAPKs are known to mediate cellular responses by phosphorylating myriad protein categories especially transcription factors. AtWRKY33 and NbWRKY8 are known targets of MAPKs induced in response to pathogen stress^4^. The transcription factors SPEECHLESS and MUTE, which regulate stomatal development, are known targets of MAPKs^8^. Apart from these, TGAs^9, 10^, ERFs^4^, bZIP transcription factors and NPR^10^ have been found to be targets of MAPKs. In *A. thaliana*, MKK and MAPK interaction and phosphorylation patterns have been elucidated by Y2H and protein microarray studies^9, 11^. The *Arabidopsis* interactome mapping consortium (http://interactome.dfci.harvard.edu/A_thaliana/) revealed ~6200 binary protein-protein interactions including some MAPK cascade members elucidating a wider repertoire of MAPK targets. Among the crop plants, MKK-MAPK interaction networks have been identified in rice^10^, pepper^12^, and cotton^13^. However, many differences have been noted in context to gene number and interaction patterns of MKK-MAPK among the studied plants. The wide variety of responses mediated by MAPKs suggests that there is more to the story than is apparent.

Legumes are central to our agricultural system for their diverse nutritional profiles and ability to fix atmospheric nitrogen by forming symbiotic relationship with soil bacteria. They are used for food (beans, lentils and peas), livestock forage or silage (alfalfa and clover) and oil production (soybean and groundnut). The 68^th^ UN general assembly declared 2016 as the international year of pulses to highlight their role as nutritious seeds for a sustainable future (www.fao.org/pulses-2016). Despite their importance, a large-scale interaction study in legume MKKs and MAPKs has not been performed till date. Only few functional studies on MAPK signalling components of legumes have been carried out^14–16^. Therefore, a robust interaction analysis between MKK-MAPKs is a prerequisite for elucidating the role of MAPK cascade members in legumes.

Hence, this study involves the integration of orthologous MKK and MAPK gene information from 16 important legume species with the aim to generate a comprehensive updated database and phylogeny of these genes in legumes. Chickpea was taken as a system to study the MKK-MAPK interaction network in legumes instead of wild and non-cultivated models like *L*. *japonicus* and *M*. *truncatula*. It is the world’s third most produced legume crop after *G. max* and *Arachis hypogea* and ranks first among pulses and the cultivated diploid legumes^17^. Many reproducible interactions were identified between 16(+5 isoforms) MAPKs and 7(+6 constitutively active isoforms) MKKs of chickpea. Further, adding to the intricacies of MAPK signalling cascades in legumes is the identification of plant-specific TCP transcription factors as novel interacting partners of chickpea MAPKs in this study. The identified network can be used as a resource in legumes to understand the relay of dynamic information through MAPK cascades, especially in legume-specific events.

## Results

### MKK and MAPK gene repertoire in legumes

In plants, MKKs and MAPKs have been mainly studied in *Arabidopsis*, rice and few solanaceae family members. Although in some legumes these genes have been identified at sequence level, reports on the interaction and functional analysis of the same are few. Recent surge in the availability of genome and transcriptome sequences for economically important legume species encouraged us to identify and develop a protein sequence-based phylogenetic relationship among MAPK cascade members. We utilized various ESTs, transcriptome shotgun assembly (TSA) and genomic sequence data sets of 16 economically important and model legumes to identify MKK and MAPK-encoding genes in them (see Supplementary Table S1). The protein sequences that resulted in a verified plant MKK and MAPK domain at NCBI conserved domain database were included. The ‘MAPK_like’ genes were excluded from this analysis. However, partial sequences that were highly similar to MKK and MAPK were included. The number of identified genes from these two functionally related groups is summarized in Table 1.

**Table 1:** Number of MKK and MAPK members identified in 16 legume species.

	Legumes	MKK groups					MAPK groups				
		A	B	C	D	Total	A	B	C	D	Total
1	*Arachis duranensis*	3	1	1	2	7	2	5	2	7	16
2	*Arachis ipaensis*	3	1	1	2	7	2	5	2	7	16
3	*Cajanus cajan*	3	1	1*	2	7	2	5	2	8	17
4	*Cicer arietinum*	3	1	1	2	7	2	5	1	8	16
5	*Glycine max*	5	2	2	3	12 + 3*	4	9 + 1*	4	14	32
6	*Lotus japonicus*	3	1	1	2	7	2	4	4	7 + 1*	18
7	*Lupinus angustifolius*	4	1	3	2	10	6	4 + 1*	4	14	29
8	*Medicago truncatula*	3	1	1	1 + 1*	7	2	5	4 + 5*	8 + 1*	25
9	*Phaseolus vulgaris*	3	1 + 1*	1	2	8	2	4	2	7	15
10	*Pisum sativum*	3	1	1	1	6	2	5	1	8	16
11	*Trifolium pratense*	3	1	1	2	7	2	5	4	8	19
12	*Trifolium subterraneum*	4	1	1	2	8	2	5	3	8	18
13	*Vicia faba*	2 + 1*	1	1	1	6	2	4	1	5 + 3*	15
14	*Vigna angularis* var. *angularis*	3	1	1	2	7	2	4	2	7	15
15	*Vigna radiata*	3	1	1	2	7	2	4	2	7	15
16	*Vigna unguiculata* subsp. *unguiculata*	3	1	1	2	7	2	4	2	6 + 1*	15

The partial sequences are marked with asterisk (*). A full-list of gene/protein identifier along with edited protein sequences is available online in Supplementary Table S2.

Monocots and dicots both have different number of genes and this number divergence is also reflected within close legumes. Allotetraploid *G. max* has the highest number of genes in both MAPK cascade groups or almost two set of paralogous genes with respect to other legumes that may also be possibly true for *A. hypogea*. However, we could identify a higher repertoire of MAPKs in *L. angustifolius*. An important aspect of our analysis was the rectification of exon-intron boundaries based on ESTs/RNAseq data for the pre-annotated genes and identification of new genes from available sequences. Of the pre-annotated genes, 12% MKK and 15% MAPK sequences were rectified. These sequences were mainly of *C*. *cajan*, *L*. *japonicus*, *P*. *sativum*, and two *Trifolium* species. The TSA/genomic sequences were analysed to identify MKK and MAPK genes in *V*. *faba* and *V*. *unguiculata*. New genes in these two MAPK cascade groups were also identified in previously annotated genomes. For legumes, diversity with context to the number of identified genes and sequence was more within MAPKs as compared to MKKs. The data of these identified genes will be shared with the respective legume sequencing groups for annotation refinement.

### Phylogeny of legume MKKs and MAPKs

The MKK and MAPK members of legumes were divided into four groups^18^ as previously reported for other model plants^19^ (Table 1). They all have typical conserved protein kinase subdomains except few partial genes. At NCBI’s conserved domain database, the group A, B and D MAPK members were annotated as plant MAPKs while C group members were shown as protein kinases only. The activation motif of TEY/TDY were conserved in MAPKs with the exception of *GLYMA*_*11G021800* encoded product, which is a partial MAPK. Nevertheless, the paralogous gene *GLYMA*_*01G222000* encodes for a full MAPK. High sequence conservation among MAPKs and MKKs were mainly due to kinase domain. The N-terminal regions in MKK members and the C-terminal regions in MAPKs that were known to be involved in interaction were also conserved with respect to type of amino acids. However, homopolymeric stretches of amino acids between the ‘D-domain’ and protein kinase domain at N-terminal of C group MKKs disrupted the homology. All orthologs of MKKs have their specific arrangement at ‘D-domain’ with cluster of positively charged amino acids and hydrophobic amino acids. At genomic level, conservation is also reflected in the number of exons within a group.

**Figure 1:**
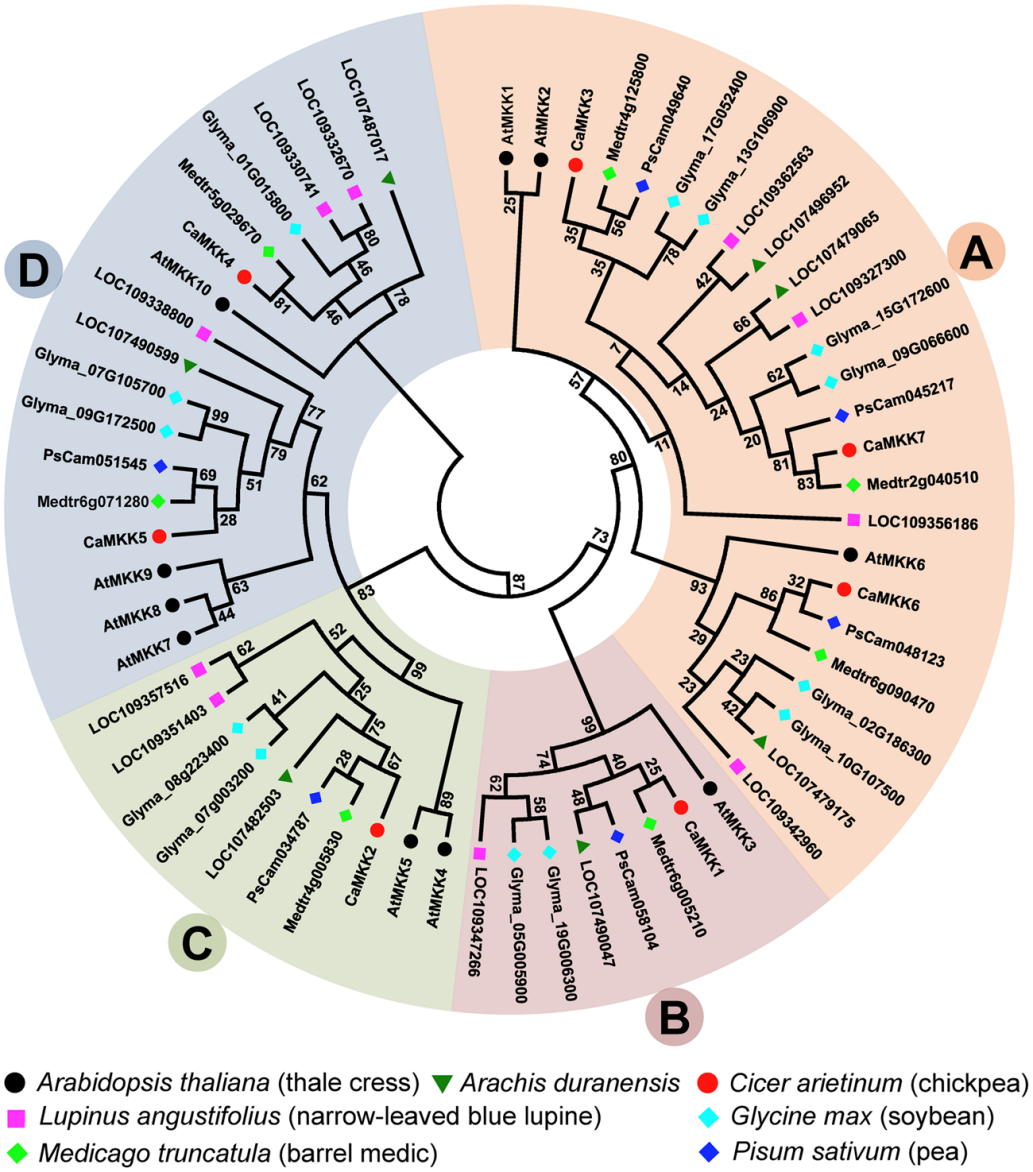
Maximum likelihood phylogeny of MKKs from six legumes and Arabidopsis. The MKKs are divided into four groups A–D. The names of the genes are indicated against deep branches. The protein sequence alignment was performed on PROMALS3D server and phylogeny was constructed using MEGA7.0.21. Phylogeny was validated by bootstrap values that were derived from 1000 iterations. A detailed phylogenetic relationship within MKKs of 16 legume species and default parameters of MEGA7.0.21 are illustrated in Supplementary Table S2.

**Figure 2:**
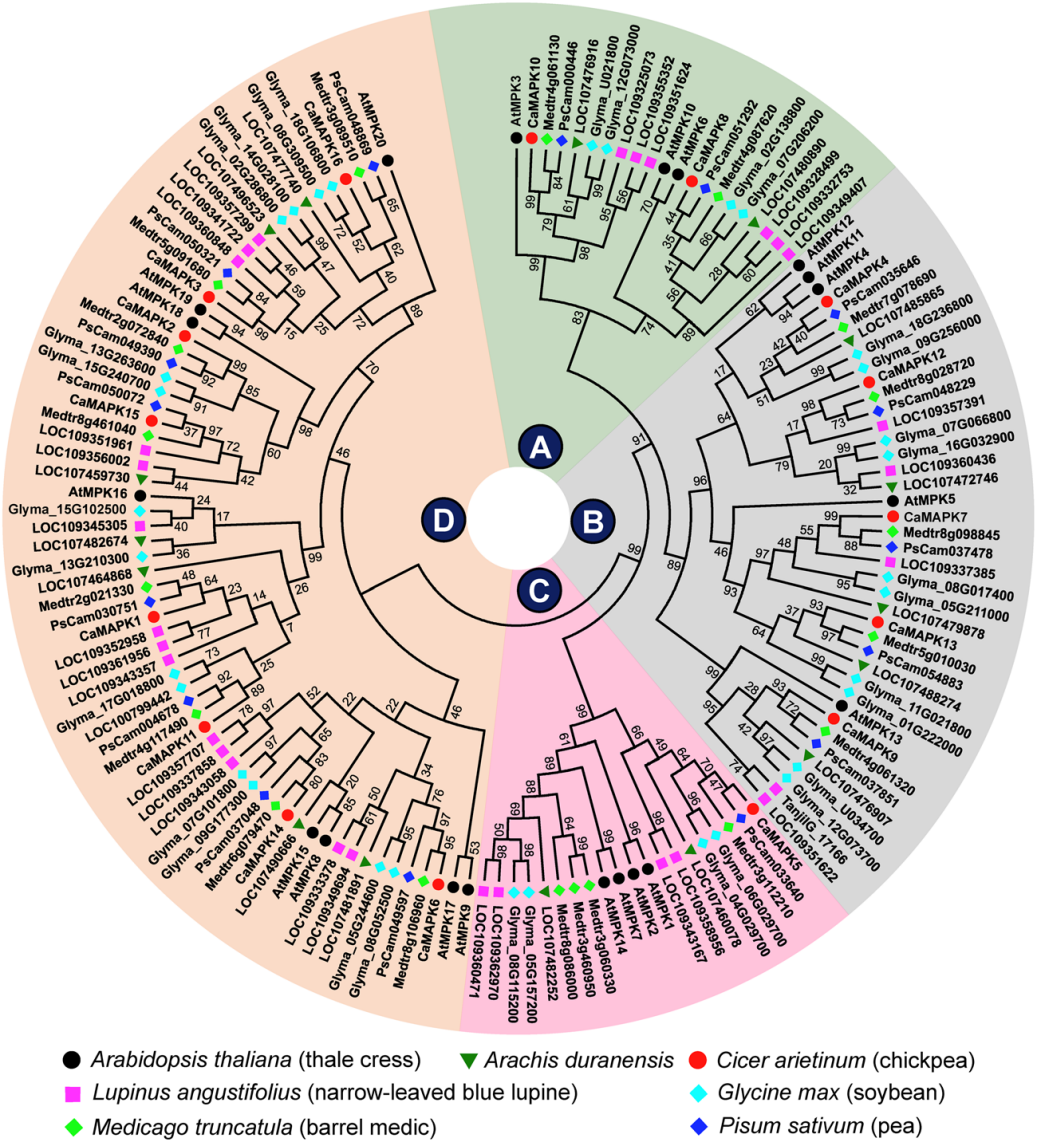
Maximum likelihood phylogeny of MAPKs from six legumes and Arabidopsis. The MAPKs are divided into four groups A–D marked at the centre. Each group is shaded with different colour for easy visualization. The protein sequence alignment was performed on PROMALS3D server and phylogeny was constructed using MEGA7.0.21 using parameters described in Supplementary Table S2 legend. Phylogeny was validated by bootstrap values that were derived from 1000 iterations. A detailed phylogenetic relationship within MAPKs of 16 legume species is illustrated in Supplementary Table S2.

The phylogeny of MKKs and MAPKs from *C*. *arietinum* and five other legumes was compared with the dicot model *A. thaliana* proteins (Figs 1 and 2). A detailed phylogenetic relationship within 16 legumes, along with edited sequences, is available online in Supplementary Table S2. In comparison to legumes, *Arabidopsis* has more members in A and D MKK groups. An interesting aspect is the divergence of AtMKK10, CaMKK4, etc members at base of D group with high confidence (bootstrap value 87%), although classically they are included within D group only (Fig. 1). This is also reciprocated by the fact that these members have unusual sequences at activation loop as compared to other MKKs. Phylogenetically, the MAPK D group is large and complex with regard to its evolution. The A and D MAPK groups have more members in *L. angustifolius* suggesting the possible evolution of new signalling cascades involving these additional members. In C group, *M*. *truncatula* has many partial MAPK genes due to tandem-duplication events while *C*. *arietinum* has only a single MAPK. In MAPK D group, a possible event of gene loss was observed in phaseoloid clade legumes (*G*. *max*, *P*. *vulgaris*, and three *Vigna* spp.) along with two *Arachis* species as *CaMAPK15* othologs are absent. In B group MAPKs, the CaMAPK4 orthologs were absent in *P*. *vulgaris* and three *Vigna* species. Our analyses have thus presented here an updated list of MKK and MAPK sequences and their easily accessible phylogeny for legume research.

### Cloning of chickpea MKKs and MAPKs

After establishing a phylogenetic relationship within legume MKKs and MAPKs, the next step was to decipher interactions between them. The position of chickpea as a diploid commercial pulse crop and availability of two independent genome sequences for *Kabuli* and *Desi* type seeds made it a very useful legume system for this interaction analysis. The MKKs and MAPKs of chickpea were analysed and numbered according to their chromosomal position. During cloning, the PCR amplified products in few MAPKs revealed the presence of multiple bands suggesting alternatively spliced isoforms. All the observed splice variants were cloned and sequenced. Spurious clones were discarded and five D group splice variants were included for further interaction studies. *CaMPK14* had two splice variants named 14-1 and 14-2. *CaMPK14*-*2* was formed as a result of exon skipping where exon 4, 5, 6, and partial 7 was absent leading to absence of full kinase domain. The second splice variant of *CaMPK6* was also found to have skipped exons, in this case exons 6 and 7 (see Supplementary Fig. S1A). The two splice variants of *CaMPK1* were due to alternative splice site selection that resulted in the difference of only three amino acids. *CaMPK3*, on the other hand had three variants formed as result of partial exon skipping. In one of them ORF was disrupted by a stop codon after kinase domain. The MAPKs, including the observed splice variants, and MKKs were cloned in the entry vector, pENTR (see Supplementary Fig. S1B) and later mobilized into newly generated split-ubiquitin based destination vectors. To make MKK-MAPK reciprocal and their intra-family interactions possible, all MAPKs and MKKs were cloned in both destination vectors.

In MAPK sequential cascade, MKKs are phosphorylated and activated by upstream kinases at two sites between subdomains VII and VIII within the conserved activation loop (S/TxxxxxS/T). Therefore, the phosphomimetic mutations can make MKKs constitutively active. Six constitutively active chickpea MKKs were generated by replacing conserved serine and threonine residues to glutamic acid (MKK^EE^). However, it was not possible for CaMKK4 since it did not have the conserved serine and threonine residues in the active site, like its *Arabidopsis* ortholog AtMKK10^9^. All chickpea entry clones generated in this study are available for research community.

### MKK-MAPK interaction network in chickpea

**Figure 3:**
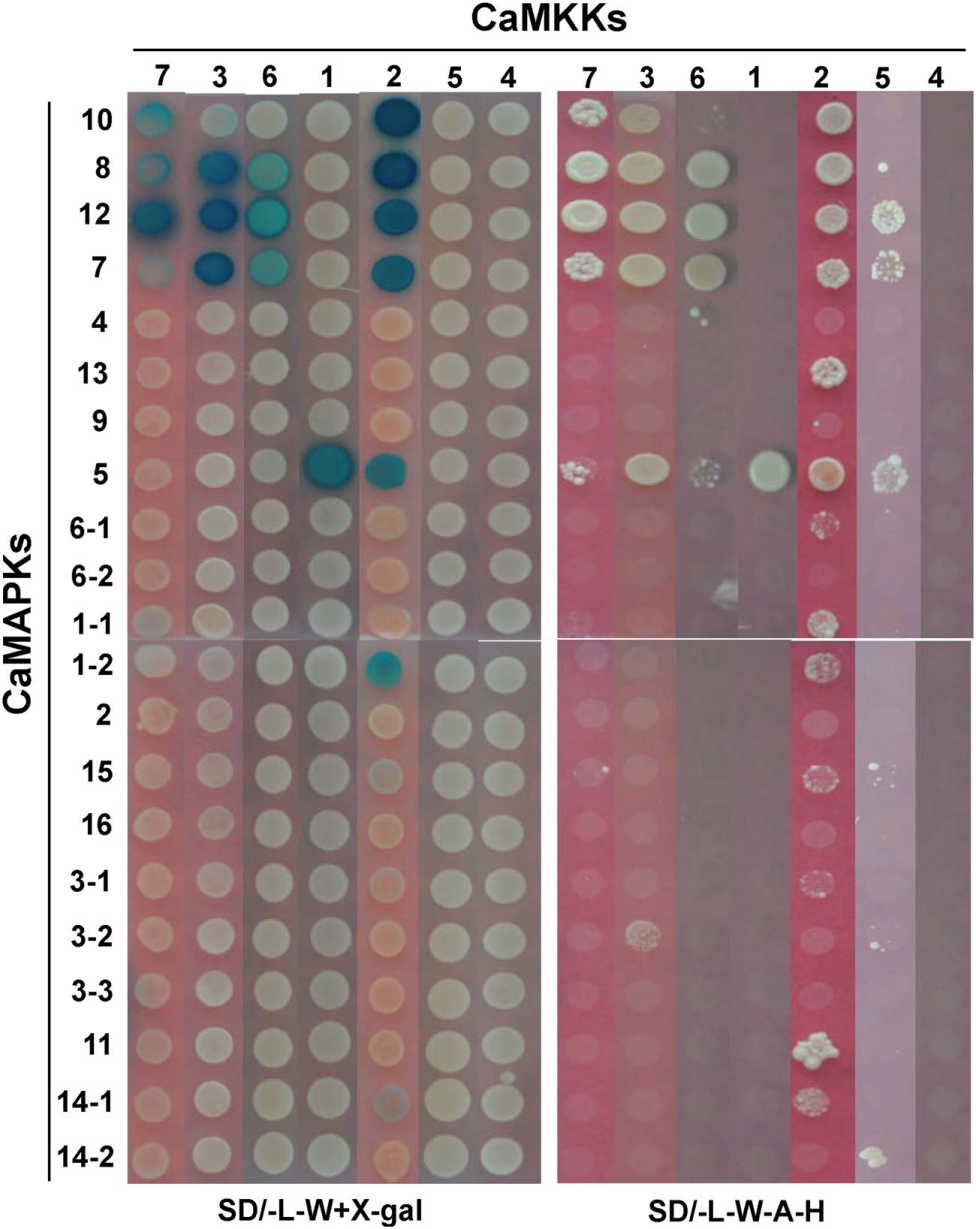
Interaction analysis of chickpea MKKs and MAPKs by yeast two-hybrid. Split-ubiquitin based Y2H system was used for checking interactions between 7 CaMKKs and 21 CaMAPKs in 147 possible combinations. The CaMKKs were cloned in pGDHB1 and CaMAPKs in pGPR3-N vector. The plates were photographed after 48 h of yeast growth. Strong protein-protein interactions showed growth on SD/-L-W-H-A media and blue colour after X-gal overlay assay. Weak interactions showed less growth on SD/-L-W-H-A media and light blue colour. Photographs were arranged in an array for comparison and better visualization. Reciprocal interaction assay was also performed.

**Figure 4:**
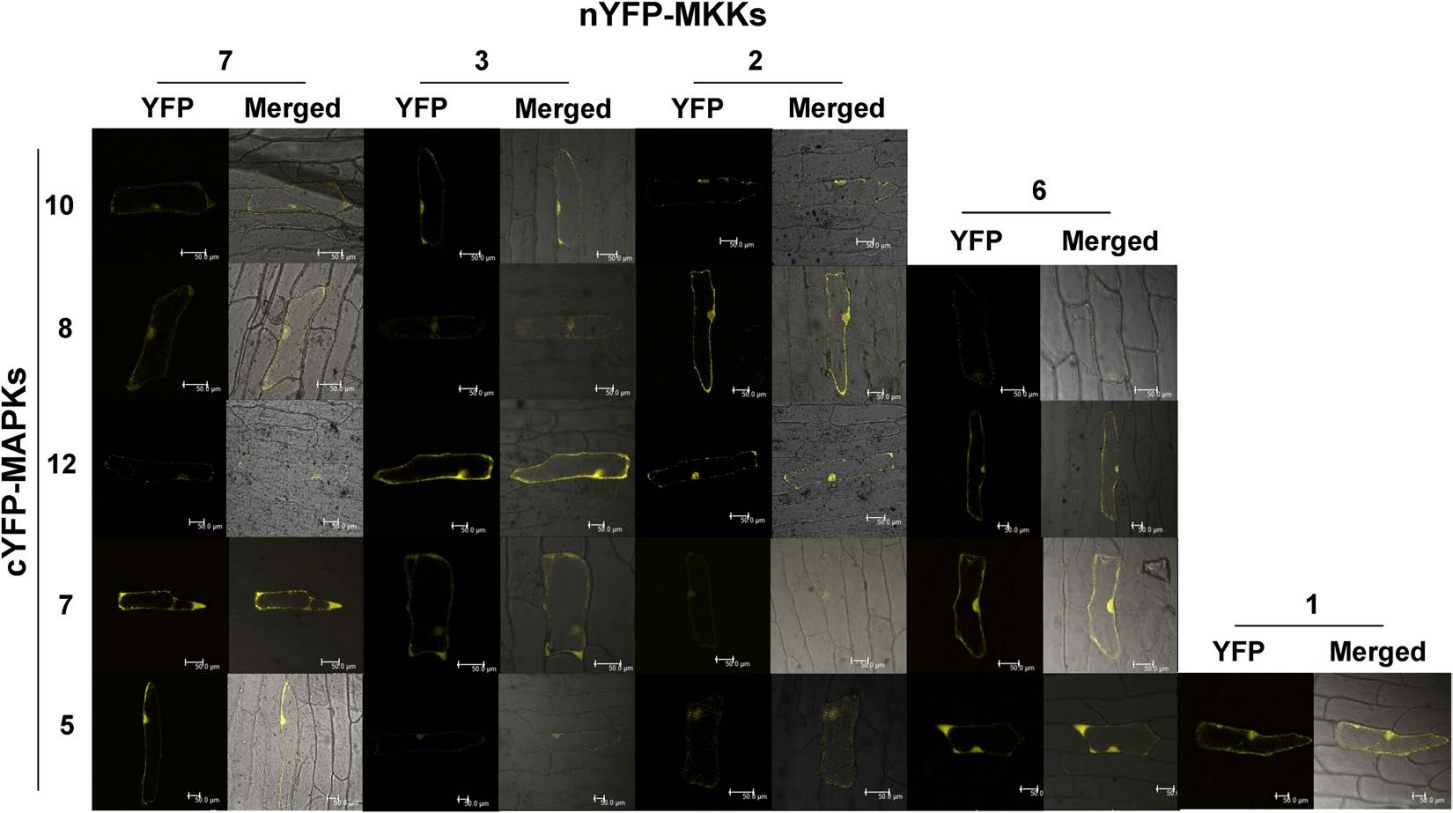
BiFC assay to verify the MKK-MAPK interactions identified by Y2H. Chickpea MKKs were tagged with N-terminal half of YFP and MAPKs were tagged with C-terminal half of YFP. Plasmid DNA of MKK and MAPK clone was precipitated on gold particles and bombarded on onion epidermal cells. Cells were observed for YFP fluorescence under a confocal microscope after 48 hours. YFP and merged (bright field and YFP) photographs are presented here for 20 interaction pairs of chickpea MKK-MAPK. BiFC assay negative controls (not shown here) were also checked. Scale bars, 50 µm.

To systematically identify MKK-MAPK interacting pairs using the mating based split-ubiquitin Y2H assay, 21 MAPKs (16 MAPKs and 5 splice variants), 7 MKKs and 6 MKK^EE^s were cloned upstream of Cub-LexA-VP16 in bait vector and transformed into NMY51 yeast strain (see Supplementary Fig. S2). Expression and correct endoplasmic reticulum membrane insertion of baits were validated by the ‘NubG/I test’^20^ to avoid false negatives (see Supplementary Fig. S3). The bait and the prey clones were also mated with mating compatible yeast strains transformed with the blank vectors in order to rule out auto-activated false positive results (see Supplementary Fig. S4). The activation of *LacZ* (checked by X-gal overlay assay) and growth on SD/-L-W-A-H plates within 48 h was considered as strong interactions in qualitative test. In contrast, the yeast growth after 48 h and weak activation of *LacZ* was considered as weak interactions. An exhaustive reciprocal mating experiments revealed a variety of interactions among all four groups of MKKs and MAPKs. All A group MKKs of chickpea interacted with MAPKs of A, B and C groups. The sole B group member, CaMKK1, interacted only with the C group CaMAPK5. The D group member, CaMKK5, showed weak interaction with CaMAPK5, CaMAPK7, and CaMAPK12 (Fig. 3). The sole C group member, CaMKK2, exhibited some interesting interaction patterns. It interacted with D group MAPKs apart from MAPKs of A, B, and C groups. CaMKK2 interacted with both the splice variants of CaMAPK1, even though the strength of the interaction was lesser in CaMAPK1-2. This interaction was observed in CaMKK2^EE^ as well. The constitutively active forms of the MKKs showed similar interaction patterns as their wild-type counterparts (see Supplementary Fig. S5). Among the two splice variants, CaMAPK14-1 interacted weakly with CaMKK2 and CaMKK2^EE^. These interactions, however, could not be detected in reciprocal possibly because of the steric hindrance among the chimeric proteins (see Supplementary Fig. S6). BiFC assays were performed in onion epidermal cells to further validate Y2H observed interactions. In these assays, MAPKs were tagged with the C-terminal YFP half and MKKs with the N-terminal half of YFP. The strong interactions of A, B, and C group MKKs were repeatedly detected by BiFC. All the interaction signals were observed in both cytoplasm and nucleus of onion epidermal cells (Fig. 4). However, the weaker interactions of CaMKK2/CaMKK2^EE^, repeatedly observed in Y2H assays, with D group MAPKs could not be verified by BiFC.

To identify possible interactions within MAPK members, Y2H was performed. Although such interactions are known in rice^10^ for MAPKs, we could not detect any such interaction within chickpea MAPK members in our experiments (data not shown). However, chickpea MKK-MKK interaction study revealed some interesting results. Both CaMKK2 and CaMKK2^EE^ were found to homo-dimerize weakly (see Supplementary Fig. S7A). CaMKK5 was found to interact strongly with CaMKK3. Weak interactions of CaMKK5 were observed with CaMKK7 and CaMKK6. Among the interactions of CaMKK5, observed in Y2H experiments, we could verify the interaction of CaMKK5 with CaMKK3. This interaction was found in both cytoplasm and nucleus of onion epidermal cells (see Supplementary Fig. S7B).

### Interaction of chickpea MAPKs with TCP transcription factors

**Figure 5:**
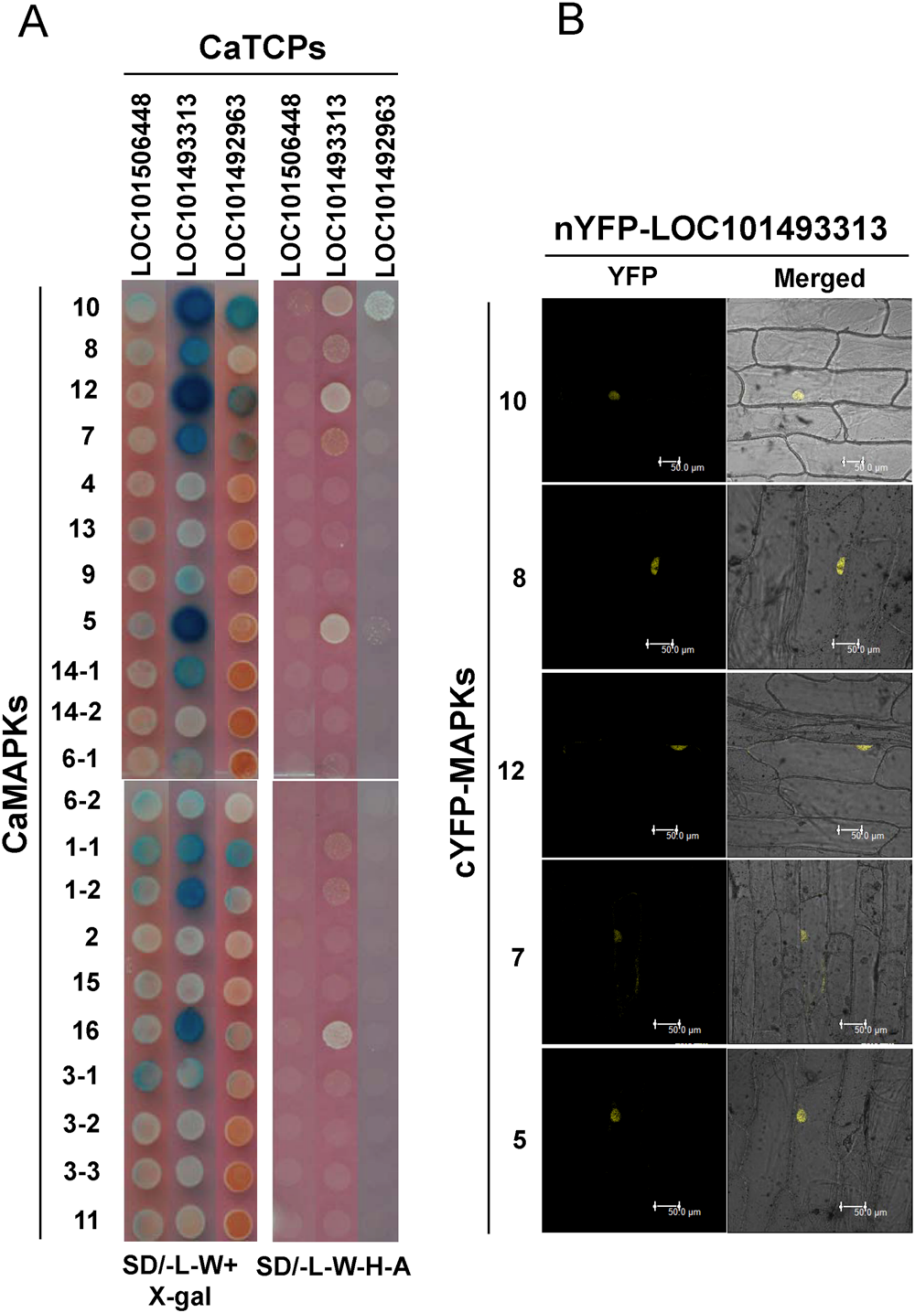
Interaction analysis of chickpea CaMAPKs with CaTCP transcription factors. (**A**) Y2H analysis of chickpea CaMAPKs with CaTCPs. Three members of class I TCP transcription factors from chickpea were fused with Cub-LexA-VP16 in pGDHB1vector and transformed in yeast strain NMY51. Chickpea MAPKs were fused with mNub in pGPR3-N vector and transformed in yeast strain NMY61. After mating and two round of selection, the yeast cells were spotted on SD/-L-W and SD/-L-W-H-A plates. Plates were photographed after 48 h growth and X-gal overlay assay was performed to check the activation of *LacZ* gene as third reporter. Yeast growth on SD/-L-W-H-A and blue colour after X-gal overlay assay suggests protein-protein interaction. (**B**) BiFC assay of five CaMAPKs and one CaTCP encoded by *LOC101493313*. Independently five CaMAPKs were tagged with C-terminal half of YFP and CaTCP was tagged with N-terminal half of YFP. Plasmid DNA of a CaMAPK and CaTCP pair was precipitated on 1 µm gold particles and bombarded on onion epidermal cells. The cells were observed under confocal microscope after 48 hours. In all five, YFP signal was visualised in nucleus. Scale bars, 50 µm.

The transcription factors are important downstream candidates for MAPK cascade as they regulate gene expression under dynamic environmental responses and many such interactions have been earlier reported. The plant-specific TCP transcription factor family is known for its role in plant growth, development, immunity, and circadian clock regulation^21, 22^. Thus, we were interested in exploring the possibility of a legume MAPK targeting TCP transcription factors and adding another layer of regulation in TCP complexes. We identified eight class I TCPs from chickpea genome using *Arabidopsis* TCPs as query and cloned six of them. However, only clones of *LOC101492963*, *LOC101493313*, and *LOC101506448* passed the NubG/I test. The interaction tests with blank vector ruled out any possible autoactivation by these three TCPs, a drawback commonly observed in GAL4 based Y2H system for transcription factors. We proceeded to check the interaction of these three TCPs with the 21 MAPK isoforms. The reciprocal mating-based Y2H interaction study with chickpea MAPKs revealed novel interactions in legumes. The *LOC101493313* encoded TCP, a homolog of AtTCP7, was found to interact strongly with group A, B, and C members while weakly with D group MAPK members (Fig. 5A). The interaction of this TCP with a D group member, CaMAPK16, was surprising since targets for D group MAPKs have rarely been reported. This TCP also showed weak interactions with both splice variants of CaMAPK1, CaMAPK14-1, and CaMAPK6-1. Qualitatively weak interactions were observed only by the activation of *LacZ* reporter gene. Another TCP encoded by *LOC1010492963* gene showed strong interaction with CaMAPK10 and weak interactions with CaMAPK12 and CaMAPK1 (Fig. 5A). The strong interactions observed in Y2H assays were confirmed *in planta* by BiFC and as expected these interactions were found to be localized in onion epidermal cell nucleus (Fig. 5B). The TCP encoded by *LOC101506448* gene failed to interact with any of the chickpea MAPKs. Our results therefore demonstrate differential interaction pattern of two TCPs with MAPKs in chickpea.

## Discussion

The emergence of complexity in MAPK cascade from early eukaryotes to plants is contributed by gene duplication events and the evolution of interaction motifs in them^23^. The role of MAPKs is not only limited to phosphorylation as they can also behave as structural adaptors^24^. Development of a MAPK cascade interactome map in legumes should therefore hasten the process of their functional characterization as seen in dicot and monocot model systems. Earlier studies and databases available for legume MAPK members have either used outdated versions or have presented the original annotated sequences without rectifying them during their analysis^25, 26^. Our MKK and MAPK gene identification, sequence rectification, and phylogeny development in legumes was the first step of this study. It provided some novel insights into the evolution of legume MAPKs. The common absence of a specific member, from the highly stable plant D group, points towards a need to revisit the relationship of *Arachis hypogea* progenitors with the five legumes of the phaseoloid clade. This analysis also points towards a problem of chimeric TSA sequences due to a highly conserved central kinase domain in these proteins. It results in wrong annotation from TSA sequences, especially in large size genomes like *L.culinaris*, *P*. *sativum*, and *V. faba*. The more number of MAPK cascade gene identification in narrow-leafed lupin (*L*. *angustifolius*) may be due to whole-genome triplication in genistoid lineage^27^. In contrast to many other legumes, a C group MAPK was absent in chickpea. We failed to identify this gene from both *Kabuli* and *Desi* sequenced genomes of chickpea. The PCR reactions using degenerate primers of *M*. *truncatula* orthologs with chickpea cDNAs were negative. Thus, we believe chickpea has lost this gene or it expresses under certain unique condition only.

**Figure 6:**
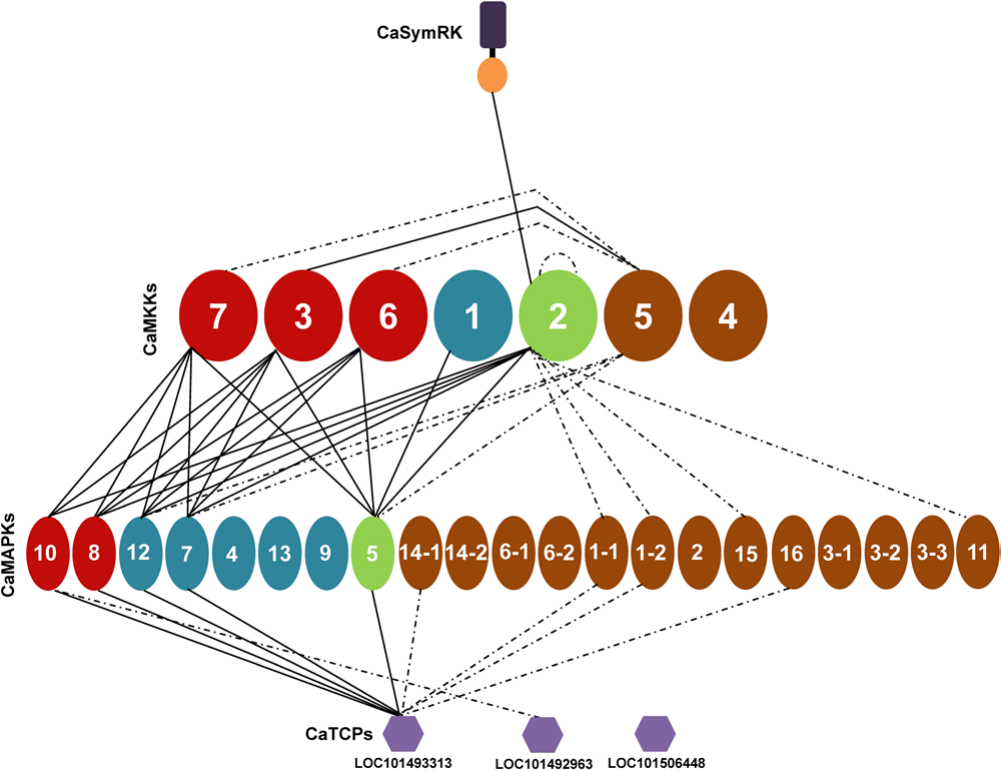
Schematic representation of identified chickpea MKK-MAPK-TCP network. The interactions observed in the study have been graphically represented. The different groups of MKKs and MAPKs have been given different shades for easier visualization. Interactions that have been identified by yeast two-hybrid and verified *in planta* by bimolecular fluorescence complementation (BiFC) are represented by solid lines while those checked through reciprocal yeast two-hybrid assays only are represented by dotted lines.

The Y2H assay is the first option to be utilised for identification of the MKK and MAPK networks^28^. Earlier the Gal4-based Y2H system has been extensively used but reciprocal interaction check in this system, with transcription factors as bait, may result in autoactivation of reporter genes^11^. Hence, we generated destination vectors of split-ubiquitin based Y2H system for large-scale binary interaction and cDNA library screenings. The LR reaction generated clones from these Y2H vectors will be compatible with *Not*I and *Asc*I restriction digestion based cloning too, adding to their versatility during cloning. We could observe some commonalities and differences between the available data from the literature and the data generated in chickpea through our study. This implies that earlier identified MAPK cascades must be operating in legumes too and new cascades emerged due to change in number of cascade genes and sequences at interaction motifs. Interactions of A group MKKs are more reported with A and B group MAPK members in individual and large-scale studies^9, 11, 29^. The celebrated MKK4/5-MPK3/6 cascade^30^ of *Arabidopsis* was observed in chickpea as well. The sole C group member of chickpea, CaMKK2, apart from interacting with CaMAPK8 and CaMAPK10, the orthologs of AtMPK6 and AtMPK3 respectively (Figs 3 and 6), interacted with two members of B group MAPKs and the only member of C group MAPK. In *Arabidopsis*, the phosphorylation of AtMPK5 by AtMKK5 is reported by Popescu *et al*.^9^. The same report shows the phosphorylation of AtMPK16 by AtMKK4/5. Similarly, chickpea CaMKK2 showed weak interactions with CaMAPK1 and CaMAPK11, the possible orthologs of AtMPK16. Even though, we observed interaction in both the splice variants of CaMAPK1, there was a slight difference in the strength of the interaction (Fig. 3). In *Arabidopsis*, AtMKK3 and three C group MPKs participate in pathogen signalling and in chickpea CaMKK1only interacts with the sole C group MAPK signifying the existence of similar cascade^31^. Another aspect in all MKK-MAPK Y2H assays is the seemingly lower number of interactions identified with the eight highly conserved D group MAPKs, having C-terminal extended region. Nevertheless, in phosphorylation assays MKKs phosphorylate D group MAPKs. This could be due to the requirement of other factors like calmodulins^32^ or scaffolding proteins that influence D group MAPKs activity and interactions *in planta*. A clear contrast with the *Arabidopsis* studies^33, 34^ was the absence of interactions from D group MKKs with A group MAPKs in chickpea (Fig. 6). The interaction of chickpea MKKs and MKK^EE^s with MAPKs were similar. This suggests that dual-phosphorylation at the activation loop do not influence their interaction preferences with MAPKs rather it influences their dual-specificity protein kinase activity. The generation of MAPK isoforms through alternative splicing may have role in differential interaction with downstream targets or relaying signals of varying quality.

The homopolymerization of MAPK members may further add to the existing complexity of MAPK signalling. In animal models, the dimerization of Mek1-Mek2 determines the strength and duration of the Erk signal^35^. To the best of our knowledge, such reports are not yet known in plant systems. Homodimer formation in chickpea MKKs is thus the first such report in plants. We observed homodimerization of CaMKK2; dimerization of CaMKK5 with CaMKK2 in yeast and in the cytoplasm and nucleus of onion epidermal cells (see Supplementary Fig. S7). It would be interesting to investigate the possible implications of such MKK dimerization events in plants.

Plant molecular biologists working on MAPK cascade are always in search of their downstream targets. Some of the important targets are still elusive. The TCP transcription factor family was found to be one such group. TCP proteins form cross-family transcription factor interactions and are considered network hubs^36^. Their importance can be visualized from another fact that pathogen effectors from three kingdoms of life target a set of TCP factors mostly belonging to class I TCPs^37^. Thus, for our analysis we considered class I TCPs of chickpea. An analysis of possible phosphorylation sites in NetPhos3 predicts threonine residues with high scores, suggesting a possible role of phosphorylation in the regulation of TCPs. In chickpea, our results showed interaction of AtMPK3/6 orthologs CaMAPK10/8 and AtMPK4/5 orthologs CaMAPK12/7 with protein encoded by TCP gene *LOC101493313*. This TCP protein also showed weak interaction with D group MAPKs. A phosphorylation event on TCP protein may modulate its interaction preferences in protein complex hubs. Thus, possibility of this TCP protein’s involvement in MAMP-mediated signalling should be further explored in legumes.

Rhizobium-legume symbiosis provides agronomical value in our cropping system. Members of the MAPK cascade have been previously indicated in legume symbiosis. A study during *Lupinus albus* and *Bradyrhizobium* interaction examined the role of MAPKs in regulation of infection and nodulation by kinase activity assay and use of MAPK cascade inhibitors^38^. However at molecular level, C group MKKs SIP2 (*Lj3g3v2040150*) is known to interact with kinase domain of symbiotic receptor kinase SymRK of *L. japonicus* and NORK of *Medicago sativa* ^39^. In our analysis, chickpea CaMKK2 also interacted with kinase domain of CaSymRK (*LOC101507037*) in a Gal4-based Y2H system (see Supplementary Fig. S8). In *L. japonicus* SIP2 RNAi hairy root lines, the infection thread formation and nodule initiation are suppressed suggesting a dual role of SIP2 in immunity and symbiosis. Barrel medic MtMKK5-MtMPK3/6 module, orthologs also identified in chickpea interacting pairs, negatively regulates the early symbiotic nodule formation^40^. However, in this recent study authors failed to identify the interaction of MtMKK5 with kinase domain of MtDMI2 that we have identified in chickpea in accordance with *L*. *japonicus* ^39^. This signifies the fact that identified chickpea interactions can also be tested in other legumes with confidence. Examination of chickpea MKK-MAPK interacting pair orthologs expression in *M*. *truncatula* (by *Medicago* Gene Expression Atlas) revealed interesting details regarding their transcript levels in various conditions and tissues including rhizobial symbiosis. The nodules have high level expression of *Medtr4g061130*, *Medtr8g028720*, and *Medtr8g098845*. Among their interacting MKK partners, two members of A group showed same level of expression in infected nodules. The coexpression of *Medicago* orthologs, for the chickpea interacting pairs, also suggests that the identified interaction pairs have biological significance. Development of RNAi and CRISPR/Cas9 methods will certainly elucidate the role of legume interacting pairs^41^.

To conclude, this work has mapped a high quality interaction network of MKKs and MAPKs in chickpea. On the basis of exhaustive comparative analysis of MKKs and MAPKs in 16 legume species, a phylogenetic relationship between them was developed. These two steps will make these interactions as reference for diverse legumes or possibly for other plant systems. Further, the identification of plant-specific TCP transcription factors as novel downstream targets of the MAPK cascade will be examined in detail for its significance. Our study on legumes is expected to augment research on the role of MAPKs in symbiosis, legume-specific roles, and other areas of plant growth and immunity.

## Methods

### Identification and *in silico* analyses of legume MKKs and MAPKs

To identify MKKs and MAPKs from legumes, BLASTp and tBLASTn were executed using *G. max* and *M. truncatula* annotated proteins as query against the assembled genome/transcriptome/annotated proteins of 16 legume species as database. The different sources that were used for analyses of the legume sequences are mentioned in Supplementary Table S1. The identified MKK and MAPK protein sequences were verified for accuracy using BLASTp at https://blast.ncbi.nlm.nih.gov/Blast.cgi. Correction for the exon-intron boundaries of coding regions was performed using the preferential order of ESTs, TSA database and nearest legume homologue sequence as reference. The exon-intron structure was generated using Gene Structure Display Server (GSDS 2.0; http://gsds.cbi.pku.edu.cn) tool. The multiple sequence alignment, phylogenetic and other *in silico* analysis was performed as earlier described by Kumar *et al*. (2016)^42^.

### Open entry clone generation and mobilization into Y2H vectors

Chickpea *MKK*, *MAPK*, and *TCP* ORFs were amplified using gene specific primers (see Supplementary Table S3). The primers were designed with sequences of the restriction enzymes *Not*I and *Asc*I on the forward and reverse sequences, respectively. In case of *CaMAPKs*, where multiple bands were observed all the bands were cloned and sequenced. Following sequencing, only those splice variants that formed a stable construct and did not harbour premature stop-codon were taken for further analysis. Thus, 16 *MAPKs* along with their five splice-variants and seven *MKKs* were amplified from chickpea Pusa 362 variety and cloned in pENTR vector. These ORFs were mobilized into the modified split-ubiquitin based Y2H destination vectors as described by Kumar *et al*.^43^. The destination vectors pGDHB1 and pGPR3-N were generated by cloning *ccd*B cassette in pDHB1 and pPR3-N vectors (Dualsystems Biotech AG, Switzerland), respectively.

### Generation of constitutively active MKKs

Phosphomimetic mutations in the active site of MKKs were induced by a PCR-based method^44^. The MKKs were amplified in two stages; in first stage, entry clone ORF halves were amplified using a M13 and a gene specific primer with mutations resulting in glutamic acid instead of serine or threonine. In second stage, fusion PCR was performed to unify these products from both halves. The final mutated ORF products were cloned in pENTR. The proper incorporation of the mutation was checked by sequencing.

### Yeast two-hybrid analysis

A mating-based split-ubiquitin Y2H approach was used. The bait clones were transformed in the yeast strain NMY51 and prey clones in the NMY61 strain using EZ yeast transformation kit (MP Biomedicals, LLC). To rule out any possible autoactivation, the control pDHB1 and pPR3-N vectors were also transformed into NMY51 and NMY61, respectively. Since the split-ubiquitin system allows for checking the bait expression, the positive (pAI-Alg5) and negative control (pDL2-Alg5) vectors were also transformed into NMY61. Bait clones in NMY51 and prey clones in NMY61 were grown overnight in the corresponding auxotrophic media and 100 µl of each culture was dispensed in a 96 DeepWell PP plate (#260252, Thermo Fisher Scientific) and centrifuged at 700 × g for 5 min. The supernatant was discarded and the pellet was re-suspended in 2.5X YPDA media (pH-5.8). The resuspended culture was allowed to grow overnight at 30 °C. After centrifugation and washing of the pellet with sterile distilled water, it was resuspended in 0.9% NaCl and spotted on double drop-out (SD/-L-W) media. The mated yeast clones were allowed to pass through two rounds of patching on SD/-L-W plates and then used for reporter gene analysis on quadruple drop-out media (SD/-L-W-A-H) and X-gal overlay assay (Supplementary Fig. S1).

### X-Gal overlay assay

Activation of the *LacZ* reporter gene was observed by X-gal overlay assay. Yeast cells were spotted on SD/-L-W plates and grown for 48 h. To 1% agarose in Z buffer; 6% of dimethylformamide, 0.1% of SDS, 0.25 mg/ml of X-gal, and 0.36% of β –mercaptoethanol were added. The mixture was poured on to the plates containing yeast cells and incubated at 30 °C in the dark till the appearance of blue color.

### Bimolecular Fluorescence Complementation (BiFC)

The open entry clones were mobilised in BiFC vectors pSAT4-DEST-n(174)EYFP-Cl (TAIR stock: CD3-1089) and pSAT5-DEST-c(175-end)EYFP-C1(B) (TAIR stock: CD3-1097) using LR clonase II enzyme mix. The MKKs and MAPKs were tagged with N-terminal and C-terminal halves of YFP, respectively. Purified plasmid DNA was precipitated onto 1 µm gold particles and bombarded using Biolistic-PDS-1000/He particle delivery system (Bio-Rad) on to onion peel cells plated on Murashige and Skoog medium (Himedia, India). After incubation of 24–48 h, onion peel cells were visualized under the 20x objective of TCS SP2 confocal laser scanning microscope (Leica, Germany).

## Electronic supplementary material

Supplementary Tables and Figures

Dataset 1

## References

[1] Nakagami H, Kiegerl S, Hirt H (2004). OMTK1, a novel MAPKKK, channels oxidative stress signalling through direct MAPK interaction. J. Biol. Chem..

[2] Kong Q (2012). The MEKK1-MKK1/MKK2-MPK4 kinase cascade negatively regulates immunity mediated by a mitogen-activated protein kinase kinase kinase in *Arabidopsis*. Plant Cell.

[3] Rodriguez MCS, Petersen M, Mundy J (2010). Mitogen-activated protein kinase signaling in plants. Annu. Rev. Plant Biol..

[4] Meng X, Zhang S (2013). MAPK cascades in plant disease resistance signaling. Annu. Rev. Phytopathol..

[5] Xu J, Zhang S (2015). Mitogen-activated protein kinase cascades signaling in plant growth and development. Trends Plant Sci..

[6] de Zelicourt A, Colcombet J, Hirt H (2016). The role of MAPK modules and ABA during abiotic stress signaling. Trends Plant Sci..

[7] Ichimura K (2002). Mitogen-activated protein kinase cascades in plants: a new nomenclature. Trends Plant Sci..

[8] Lampard GR, Lukowitz W, Ellis BE, Bergmann DC (2009). Novel and expanded roles for MAPK signalling in *Arabidopsis* stomatal cell fate revealed by cell type-specific manipulations. Plant Cell.

[9] Popescu S (2009). MAPK target networks in *Arabidopsis thaliana* revealed using functional protein microarrays. Genes Dev..

[10] Singh R (2012). Rice mitogen-activated protein kinase interactome analysis using the yeast two-hybrid system. Plant Physiol..

[11] Lee JS, Huh KW, Bhargava A, Ellis BE (2008). Comprehensive analysis of protein-protein interactions between *Arabidopsis* MAPKs and MAPK kinases helps define potential MAPK signalling modules. Plant Signal. Behav..

[12] Liu Z (2015). Genome-wide identification and transcriptional expression analysis of mitogen-activated protein kinase and mitogen-activated protein kinase kinase genes in *Capsicum annuum*. Front. Plant Sci..

[13] Zhang X (2016). Integration analysis of MKK and MAPK family members highlights potential MAPK signaling modules in cotton. Sci. Rep..

[14] Daxberger A (2007). Activation of members of a MAPK module in β-glucan elicitor-mediated non-host resistance of soybean. Planta.

[15] Liu JZ (2011). Soybean homologs of MPK4 negatively regulate defense responses and positively regulate growth and development. Plant Physiol..

[16] Liu JZ (2014). Positive and negative roles for soybean MPK6 in regulating defense responses. Mol. Plant Microbe Interact..

[17] Daryanto S, Wang L, Jacinthe PA (2015). Global synthesis of drought effects on food legume production. PLoS One.

[18] Hamel LP (2006). Ancient signals: comparative genomics of plant MAPK and MAPKK gene families. Trends Plant Sci..

[19] Mohanta TK (2015). Identification of new members of the MAPK gene family in plants shows diverse conserved domains and novel activation loop variants. BMC Geno..

[20] Yao Z (2017). A global analysis of the receptor tyrosine kinase-protein phosphatase interactome. Mol. Cell.

[21] Lopez JA, Sun Y, Blair PB, Mukhtar MS (2015). TCP three-way handshake: linking developmental processes with plant immunity. Trends Plant Sci..

[22] Wu JF (2016). LWD-TCP complex activates the morning gene *CCA1* in *Arabidopsis*. Nat. Commun..

[23] Doczi R, Okresz L, Romero AE, Paccanaro A, Bogre L (2012). Exploring the evolutionary path of plant MAPK networks. Trends Plant Sci..

[24] Edmunds JW, Mahadevan LC (2004). MAP kinases as structural adaptors and enzymatic activators in transcription complexes. J. Cell Sci..

[25] Neupane A, Nepal MP, Benson BV, MacArthur KJ, Piya S (2013). Evolutionary history of mitogen-activated protein kinase (MAPK) genes in *Lotus*, *Medicago*, and *Phaseolus*. Plant Signal. Behav..

[26] Zheng Y (2016). iTAK: A program for genome-wide prediction and classification of plant transcription factors, transcriptional regulators, and protein kinases. Mol. Plant.

[27] Hane, J. K. *et al*. A comprehensive draft genome sequence for lupin (*Lupinus angustifolius*), an emerging health food: insights into plant–microbe interactions and legume evolution. *Plant Biotechnol*. *J*. **15**, 318–330 (2017). 10.1111/pbi.12615 PMC5316927 27557478

[28] Zhang T, Chen S, Harmon AC (2016). Protein-protein interactions in plant mitogen-activated protein kinase cascades. J. Exp. Bot..

[29] Meszaros T (2006). The Arabidopsis MAP kinase kinase MKK1 participates in defense responses to the bacterial elicitor flagellin. Plant J..

[30] Asai T (2002). MAP kinase signalling cascade in *Arabidopsis* innate immunity. Nature.

[31] Doczi R (2007). The *Arabidopsis* mitogen-activated protein kinase kinase MKK3 is upstream of group C mitogen-activated protein kinases and participates in pathogen signalling. Plant Cell.

[32] Takahashi F, Mizoguchi T, Yoshida R, Ichimura K, Shinozaki K (2011). Calmodulin-dependent activation of MAP kinase for ROS homeostasis in *Arabidopsis*. Mol. Cell.

[33] Xu J (2008). Activation of MAPK kinase 9 induces ethylene and camalexin biosynthesis and enhances sensitivity to salt stress in Arabidopsis. J. Biol. Chem..

[34] Andreasson E, Ellis B (2010). Convergence and specificity in the *Arabidopsis* MAPK nexus. Trends Plant Sci..

[35] Catalanotti F (2009). A Mek1-Mek2 heterodimer determines the strength and duration of the Erk signal. Nat. Struct. Mol. Biol..

[36] Bemer M, van Dijk ADJ, Immink RGH, Angenent GC (2017). Cross-family transcription factor interactions: An additional layer of gene regulation. Trends Plant Sci..

[37] Weßling R (2014). Convergent targeting of a common host protein-network by pathogen effectors from three kingdoms of life. Cell Host Microbe.

[38] Fernandez-Pascual M (2006). Involvement of mitogen-activated protein kinases in the symbiosis. Bradyrhizobium-Lupinus. J. Exp. Bot..

[39] Chen T (2012). A MAP kinase kinase interacts with SymRK and regulates nodule organogenesis in *Lotus japonicus*. Plant Cell.

[40] Ryu H, Laffont C, Frugier F, Hwang I (2017). MAP Kinase-mediated negative regulation of symbiotic nodule formation in *Medicago truncatula*. Mol. Cells.

[41] Wang L (2016). Efficient inactivation of symbiotic nitrogen fixation related genes in *Lotus japonicus* using CRISPR-Cas9. Front. Plant Sci..

[42] Kumar K (2016). WRKY domain-encoding genes of a crop legume chickpea (*Cicer arietinum*): Comparative analysis with *Medicago truncatula* WRKY family and characterization of group-III gene(s). DNA Res..

[43] Kumar K, Yadav S, Purayannur S, Verma PK (2013). An alternative approach in Gateway^®^ cloning when the bacterial antibiotic selection cassettes of the entry clone and destination vector are the same. Mol. Biotechnol..

[44] Patel DH, Wi SG, Bae HJ (2009). Modification of overlap extension PCR: A mutagenic approach. Indian J. Biotechnol..

