# Supplementary material for: Phylogenomic analysis of MKKs and MAPKs from 16 legumes and detection of interacting pairs in chickpea divulge MAPK signalling modules
Source: Sci Rep. 2017 Jul 10;7:5026. doi: 10.1038/s41598-017-04913-0 (PMC5504024; doi:10.1038/s41598-017-04913-0)
Supplement: Supplementary file 1 — Supplementary Tables and Figures [file 41598_2017_4913_MOESM1_ESM.pdf]

Title

Phylogenomic analysis of MKKs and MAPKs from 16 legumes and detection of interacting pairs in chickpea divulge MAPK signalling modules

Authors:

Savithri Purayannur[1, 3], Kamal Kumar[1, 3], Vemula Chandra Kaladhar[2, 3], Praveen Kumar Verma[1, 4]

[1]Plant Immunity Laboratory, National Institute of Plant Genome Research, Aruna Asaf Ali Marg, New Delhi-110067, INDIA

[2]School of Life Sciences, Central University of Gujarat, Gandhinagar-382030, Gujarat, INDIA

[3]Authors have equally contributed to this work.

[4]Corresponding author

Supplementary Table S1. Genome, transcript sequence assembly (TSA) and annotated sequence resources used for mining legume MKKs and MAPKs. The available data at NCBI and Phytozome 11 was also used.

S. No.	Legumes	Resources	References
1	*Arachis duranensis*	Bioproject ID:PRJNA258023, PRJNA316327<br>http://legumeinfo.org/home<br>http://www.peanutbase.org/download	Bertioli et al., 2016
2	*Arachis ipaensis*	Bioproject ID:PRJNA258025, PRJNA316874<br>http://www.peanutbase.org/download	
3	*Cajanus cajan*	Bioproject ID: PRJNA72815	Varshney et al., 2012
		Bioproject ID: PRJNA68667	Singh et al., 2012
4	*Cicer arietinum*	Bioproject ID: PRJNA190909	Varshney et al., 2013
		Bioproject ID:PRJNA78951	Parween et al., 2015
5	*Glycine max*	Bioproject ID:PRJNA19861	Schmutz et al., 2010
6	*Lotus japonicus*	http://www.kazusa.or.jp/lotus/	Sato et al., 2008
7	*Lupinus angustifolius*	http://legumeinfo.org/home<br>BioProject ID: PRJNA356456; PRJNA299755<br>http://www.lupinexpress.org	Hane et al., 2016
		Bioproject ID: PRJNA179231	Yang et al., 2013
		Bioproject ID: PRJNA248164	Kamphuis et al., 2015
8	*Medicago truncatula*	http://jcvi.org/medicago/<br>Bioproject ID: RJNA30099	Tang et al., 2014;<br>Young et al., 2011
9	*Phaseolus vulgaris*	http://denovo.cnag.cat/genomes/bean/<br>Bioproject ID: PRJNA41439	Schmutz et al., 2014
		Bioproject ID: PRJNA221782	Vlasova et al., 2016
10	*Pisum sativum*	Bioproject ID:PRJNA211622	Duarte et al., 2014
		Bioproject ID:PRJNA277074, PRJNA277076	Sudheesh et al., 2015
		Bioproject ID:PRJNA284856	Zhukov et al., 2015
		http://bios.dijon.inra.fr/files/Peptides_PsUniLowCopy.fa	Alves-Carvalho et al., 2015
11	*Trifolium pratense*	http://legumeinfo.org/home<br>Bioproject ID: PRJNA219226	Vega et al., 2015
12	*Trifolium subterraneum*	http://clovergarden.jp/index.html<br>Bioproject ID: PRJDB2012	Hirakawa et al., 2016
13	*Vicia faba*	TSA (Bioproject ID: PRJNA225881)	Webb et al., 2016
		https://www.coolseasonfoodlegume.org/	
14	*Vigna angularis* var. *angularis*	Bioproject ID: PRJDB3778	Sakai et al., 2015
		Bioproject ID: PRJNA261643	Yang et al., 2015
		Bioproject ID: PRJNA210126, PRJNA253346	Kang et al., 2015
		http://viggs.dna.affrc.go.jp	
15	*Vigna radiata*	TSA (Bioproject ID: PRJNA260767)	Li et al., 2015
		Bioproject ID: PRJNA243847	Kang et al., 2014
16	*Vigna unguiculata*	Bioproject ID: PRJNA325510	
		Bioproject ID: PRJNA290938	Tan et al., 2016

Alves-Carvalho, S., Aubert, G., Carrère, S., Cruaud, C., Brochot, A.-L., Jacquin, F., Klein, A. *et al.* (2015) Full-length *de novo* assembly of RNA-seq data in pea (*Pisum sativum* L.) provides a gene expression atlas and gives insights into root nodulation in this species. Plant J. 84, 1–19.

Bertioli DJ, Cannon SB, Froenicke L, *et al.* 2016. The genome sequences of *Arachis duranensis* and *Arachis ipaensis*, the diploid ancestors of cultivated peanut. Nature Genetics **48**, 438-446.

Duarte J, Riviere N, Baranger A, *et al*. 2014.Transcriptome sequencing for high throughput SNP development and genetic mapping in Pea. BMC Genomics **15**, 126.

Hane JK, Ming Y, Kamphuis LG, *et al.* 2016. A comprehensive draft genome sequence for lupin (*Lupinus angustifolius*), an emerging health food: insights into plant–microbe interactions and legume evolution. Plant Biotechnology Journal, doi:10.1111/pbi.12615.

Hirakawa H, Kaur P, Shirasawa K, Nichols P, Nagano S, Appels R, Erskine W, Isobe SN. 2016. Draft genome sequence of subterranean clover, a reference for genus *Trifolium*. Scientific Reports **6**, 30358.

Kamphuis LG, Hane JK, Nelson MN, Gao L, Atkins CA, Singh KB. 2015. Transcriptome sequencing of different narrow-leafed lupin tissue types provides a comprehensive uni-gene assembly and extensive gene-based molecular markers. Plant Biotechnology Journal **13,** 14-15.

Kang YJ, Kim SK, Kim MY, *et al.* 2014. Genome sequence of mungbean and insights into evolution within *Vigna* species. Nature Communications**5**, 5443.

Kang YJ, Satyawan D, Shim S, *et al*. 2015. Draft genome sequence of adzuki bean, *Vigna angularis*. Scientific Reports **5,** 8069.

Li SW, Shi RF, Leng Y. 2015. *De novo* characterization of the Mung Bean transcriptome and transcriptomic analysis of adventitious rooting in seedlings using RNA-Seq. PLoS One **10**, e0132969.

Parween S, Nawaz K, Roy R, *et al.* 2015. An advanced draft genome assembly of a desi type chickpea (*Cicer arietinum* L.). Scientific Reports **5,** 12806.

Sakai H, Naito K, Ogiso-Tanaka E, Takahashi *et al*. 2015. The power of single molecule real-time sequencing technology in the *de novo* assembly of a eukaryotic genome. Scientific Reports **5,** 16780.

Sato S, Nakamura Y, Kaneko T, *et al*. 2008. Genome structure of the legume, *Lotus japonicus*. DNA Research **15**, 227-239.

Schmutz J, Cannon SB, Schlueter J, *et al*. 2010. Genome sequence of the palaeopolyploid soybean. Nature **463**, 178-183.

Schmutz J, McClean PE, Mamidi S, *et al.* 2014. A reference genome for common bean and genome-wide analysis of dual domestications. Nature Genetics **46**, 707-713.

Singh NK, Gupta DK, Jayaswal PK, *et al*. 2012. The first draft of the pigeonpea genome sequence. Journal of Plant Biochemistry and Biotechnology **21**, 98-112.

Sudheesh S, Sawbridge TI, Cogan NO, Kennedy P, Forster JW, Kaur S. 2015. *De novo* assembly and characterisation of the field pea transcriptome using RNA-Seq. BMC Genomics **16**, 611.

Tan H, Huang H, Tie M, Tang Y, Lai Y, Li H. 2016. Transcriptome profiling of two Asparagus bean (*Vigna unguiculata* subsp. *sesquipedalis*) cultivars differing in chilling tolerance under cold stress. PLoS ONE **11**, e0151105.

Tang H, Krishnakumar V, Bidwell S, *et al*. 2014. An improved genome release (version Mt4.0) for the model legume *Medicago truncatula*. BMC Genomics **15**, 312.

Varshney RK, Chen W, Li Y, *et al.* 2012. Draft genome sequence of pigeonpea (*Cajanus cajan*), an orphan legume crop of resource-poor farmers. Nature Biotechnology **30,** 83-89.

Varshney RK, Song C, Saxena RK, *et al*. 2013. Draft genome sequence of chickpea (*Cicer arietinum*) provides a resource for trait improvement. Nature Biotechnology **31**, 240-246.

Vega JJD, Ayling S, Hegarty M, *et al.* 2015. Red clover (*Trifolium pratense* L.) draft genome provides a platform for trait improvement. Scientific Reports **5,** 17394.

Vlasova A, Capella-Gutierrez S, Rendon-Anaya M, *et al*. 2016. Genome and transcriptome analysis of the Mesoamerican common bean and the role of gene duplications in establishing tissue and temporal specialization of genes. Genome Biology **17,** 32.

Webb A, Cottage A, Wood T, *et al*. 2016. A SNP-based consensus genetic map for synteny-based trait targeting in faba bean (*Vicia faba* L.). Plant Biotechnology Journal *14*, 177-185.

Yang H, Tao Y, Zheng Z, *et al.* 2013. Draft genome sequence, and a sequence-defined genetic linkage map of the legume crop species *Lupinus angustifolius* L. Plos One **8**, e64799.

Yang K, Tian Z, Chen C, *et al*. 2015. Genome sequencing of adzuki bean (*Vigna angularis*) provides insight into high starch and low fat accumulation and domestication. Proceedings of the National Academy of Sciences USA **112**, 13213-13218.

Yates SA, Swain MT, Hegarty MJ, *et al.* 2014. *De novo* assembly of red clover transcriptome based on RNA-Seq data provides insight into drought response, gene discovery and marker identification. BMC Genomics **15**, 453.

Young ND, Debelle F, Oldroyd GED, *et al*. 2011. The *Medicago* genome provides insight into the evolution of rhizobial symbioses. Nature **480**, 520-524.

Zhukov VA, Zhernakov AI, Kulaeva OA, Ershov NI, Borisov AY, Tikhonovich IA. 2015. *De Novo* assembly of the Pea (*Pisum sativum* L.) nodule transcriptome. International Journal of Genomics **2015**, 695947.

Supplementary Table S3. List of primers used in this study. The restriction site sequences are highlighted: *Not*I with red and *Asc*I with blue.

Name	Oligonucleotide sequence	Purpose
A1MPKF	5'GCAGGCTCCGCGGCCGCCATGGCCGGAGTTAATCAAAACG 3'	Forward Primers for cloning of chickpea MAPKs in pENTR at *Not*I restriction site
A2MPKF	5'GCAGGCTCCGCGGCCGCCATGGATGGAGAAGGAGGAGC 3'	
B1MPKF	5'GCAGGCTCCGCGGCCGCCATGTCTGTTGAATCAAGTGAG 3'	
B2MPKF	5'GCAGGCTCCGCGGCCGCCATGGCAACAAAAGAGTCAAACTC 3'	
B3MPKF	5'GCAGGCTCCGCGGCCGCCATGTCTCATAACTCAGATGACAAC 3'	
B4MPKF	5'GCAGGCTCCGCGGCCGCCATGGGTAGCAAAAGCAAAGATAC 3'	
B5MPKF	5'GCAGGCTCCGCGGCCGCCATGGAAAACAACACTGAATCTGAG 3'	
C1MPKF	5'GCAGGCTCCGCGGCCGCCATGGCGACTCCGGTTGAACCAC 3'	
D1MPKF	5'GCAGGCTCCGCGGCCGCCATGGGTGGTGGAGGTACACTC 3'	
D2MPKF	5'GCAGGCTCCGCGGCCGCCATGGGTGGAGGAGGAACAATAG 3'	
D3MPKF	5'GCAGGCTCCGCGGCCGCCATGCATCCTGATCAGAGAAAAAAG 3'	
D4MPKF	5'GCAGGCTCCGCGGCCGCCATGCAGAAAGATCAACTCAAGAAG 3'	
D5MPKF	5'GCAGGCTCCGCGGCCGCCATGACGCAGAAAATTCAGCTCAAG 3'	
D6MPKF	5'GCAGGCTCCGCGGCCGCCATGCAGCAAGATCATAGGAAAAAG 3'	
D7MPKF	5'GCAGGCTCCGCGGCCGCCATGGAGAAAGGAAAGAAATCAATG 3'	
D8MPKF	5'GCAGGCTCCGCGGCCGCCATGCCTCCTGATCAGAGAAAAAAG 3'	
A1CaMPKR	5'AGCTGGGTCGGCGCGCCCTTAAGCATACTCAGGATTGAGTGC 3'	Reverse closed reading frame primers for cloning chickpea MAPKs in pENTR at *Asc*I restriction site
A2CaMPKR	5'AGCTGGGTCGGCGCGCCCCTACTGCTGATACTCAGGGTTAAATG 3'	
B1CaMPKR	5'AGCTGGGTCGGCGCGCCCTCAGTGAATTGGTGGATCAGGATTG 3'	
B2CaMPKR	5'AGCTGGGTCGGCGCGCCCTTACTGACAGGGTGGATCCGG 3'	
B3CaMPKR	5'AGCTGGGTCGGCGCGCCCTCAATAAATAGGTGGATCAGGATTG 3'	
B4CaMPKR	5'AGCTGGGTCGGCGCGCCCTTATTGAGAGAGTGGATCCGGATTG 3'	
B5CaMPKR	5'AGCTGGGTCGGCGCGCCCCTATTCCAATATCTGCTCCAGGCTG 3'	
C1CaMPKR	5'AGCTGGGTCGGCGCGCCCTCAAGAGCATATCTCTGCATTTTCC 3'	
D1CaMPKR	5'AGCTGGGTCGGCGCGCCCCTAAGCAAGGAGAGCAGCCACC 3'	
D2CaMPKR	5'AGCTGGGTCGGCGCGCCCCTAAGCATGGAGAGCAAGAATTTTATTG 3'	
D3CaMPKR	5'AGCTGGGTCGGCGCGCCCTCAGTACCACTGGCCACCTG 3'	
D4CaMPKR	5'AGCTGGGTCGGCGCGCCCCTATGACAATCCATATTGAAAGCCTG 3'	
D5CaMPKR	5'AGCTGGGTCGGCGCGCCCCTAAAACAACCCATACTGAATGCCTC 3'	
D6CaMPKR2	5'AGCTGGGTCGGCGCGCCCCTAGAACATTCTTGTCATACCGTATTG 3'	
D7CaMPKR	5'AGCTGGGTCGGCGCGCCCTCAATACTTATGGTTAAATCTGATGCTATT	
D8CaMPKR	5'AGCTGGGTCGGCGCGCCCCTACCAGTTACCGCCAGCTCC 3'	
A1CaMPKOR	5'AGCTGGGTCGGCGCGCCCAGCATACTCAGGATTGAGTGC 3'	Reverse open reading frame primers for cloning chickpea MAPKs in pENTR at *Asc*I restriction site
A2CaMPKOR	5'AGCTGGGTCGGCGCGCCCCTGCTGATACTCAGGGTTAAATG 3'	
B1CaMPKOR	5'AGCTGGGTCGGCGCGCCCGTGAATTGGTGGATCAGGATTG 3'	
B2CaMPKOR	5'AGCTGGGTCGGCGCGCCCCTGACAGGGTGGATCCGG 3'	
B3CaMPKOR	5'AGCTGGGTCGGCGCGCCCATAAATAGGTGGATCAGGATTG 3'	
B4CaMPKOR	5'AGCTGGGTCGGCGCGCCCTTGAGAGAGTGGATCCGGATTG 3'	
B5CaMPKOR	5'AGCTGGGTCGGCGCGCCCTTCCAATATCTGCTCCAGGCTG 3'	
C1CaMPKOR	5'AGCTGGGTCGGCGCGCCCAGAGCATATCTCTGCATTTTCC 3'	
D1CaMPKOR	5'AGCTGGGTCGGCGCGCCCAGCAAGGAGAGCAGCCACC 3'	
D2CaMPKOR	5'AGCTGGGTCGGCGCGCCCAGCATGGAGAGCAAGAATTTTATTG 3'	
D3CaMPKOR	5'AGCTGGGTCGGCGCGCCCGTACCACTGGCCACCTG 3'	
D4CaMPKOR	5'AGCTGGGTCGGCGCGCCCTGACAATCCATATTGAAAGCCTG 3'	
D5CaMPKOR	5'AGCTGGGTCGGCGCGCCCAAACAACCCATACTGAATGCCTC 3'	
D6CaMPKOR	5'AGCTGGGTCGGCGCGCCCGAACATTCTTGTCATACCGTATTG 3'	
D7CaMPKOR	5'AGCTGGGTCGGCGCGCCCATACTTATGGTTAAATCTGATGCTATTTC 3'	
D8CaMPKOR	5'AGCTGGGTCGGCGCGCCCCCAGTTACCGCCAGCTCC 3'	
A1MEKF	5'GCAGGCTCCGCGGCCGCCATGAAGAGAGGATGTTTAGTTCC 3'	Forward

A2MEKF	5'GCAGGCTCCGCGGCCGCCATGACCAAAGGAAACTTGGGC 3'	Primers for cloning of chickpea MKKs in pENTR at *Not*I restriction site
A3MEKF	5'GCAGGCTCCGCGGCCGCCATGAAAACCAAGACGCCATTGAAG 3'	
B1MEKF	5'GCAGGCTCCGCGGCCGCCATGTCTGGGTTAGAGGAATTGAG 3'	
C1MEKF	5'GCAGGCTCCGCGGCCGCCATGAGGCCGATTCAACTTCCAC 3'	
D1MEKF	5'GCAGGCTCCGCGGCCGCCATGGCGCTCGTCCACCGCCG 3'	
D2MEKF	5'GCAGGCTCCGCGGCCGCCATGACATTGGTTATCAGAGAAAGAAG 3'	
A1CaMEKR	5'AGCTGGGTCGGCGCGCCCTTATATGGTTGCAAGTGTAGACCCTG 3'	Reverse Primers for cloning chickpea MKKs in pENTR at *Asc*I restriction site
A2CaMEKR	5'AGCTGGGTCGGCGCGCCCTTATAAGGTTGCGAGTGGAGATCC 3'	
A3CaMEKR	5'AGCTGGGTCGGCGCGCCCTCATCTGGTAAAATTTATAGGAGGTTC 3'	
B1CaMEKR	5'AGCTGGGTCGGCGCGCCCTTATTGGCTAATATACAGTTCTTGTTTAAC 3'	
C1CaMEKR	5'AGCTGGGTCGGCGCGCCCTTAAGAAGAAAGAGGCCTTGGTGG 3'	
D1MEKR	5'AGCTGGGTCGGCGCGCCCTTAACAAGTTTCTACATCATTAC 3'	
D2MEKR	5'AGCTGGGTCGGCGCGCCCTCACCCACATAGAACATAATCCTC3'	
A1MEKOR	5'AGCTGGGTCGGCGCGCCCTATGGTTGCAAG 3'	Reverse open reading frame cloning of chickpea MKKs in pENTR at *Asc*I restriction site
A2MEKOR	5'AGCTGGGTCGGCGCGCCCTAAGGTTGCGAG 3'	
A3MEKOR	5'AGCTGGGTCGGCGCGCCCTCTGGTAAAATTTATAG 3'	
B1MEKOR	5'AGCTGGGTCGGCGCGCCCTTGGCTAATATAC 3'	
C1MEKOR	5'AGCTGGGTCGGCGCGCCCAGAAGAAAGAGG 3'	
D1MEKOR	5'AGCTGGGTCGGCGCGCCCACAAGTTTCTAC 3'	
D2MEKOR	5'AGCTGGGTCGGCGCGCCCCCCACATAGAAC 3'	
EEA1MEKF	5'GAGTCTGGTCAAGCAAATGAGTACATTGG 3'	Forward and reverse primers for the generation of constitutively active chickpea MKKs
EEA1MEKR	5'CTCATTTGCTTGACCAGACTCAGTATCC 3'	
EEA2MEKF	5'GAATCTGGTCAAGCAAATGAGTTCATTGG 3'	
EEA2MEKR	5'CTCATTTGCTTGACCAGATTCACTTTCC 3'	
EEA3MEKF	5'GAAATGGGCCAAAGAGATGAATTTGTTGG 3'	
EEA3MEKR	5'TTCATCTCTTTGGCCCATTTCACTAGCC 3'	
EEB1MEKF	5'GAAGTTGCAATGTGTGCTGAGTTCGTTGG 3'	
EEB1MEKR	5'CTCAGCACACATTGCAACTTCACTCTCTAAG 3'	
EEC1MEKF	5'GAGATGGATCCGTGCAATGAATCGGTTGG 3'	
EEC1MEKR	5'TTCATTGCACGGATCCATCTCTTGATTC 3'	
EED1MEKF	5'GAGCTTGAAGCGTGTAACGAGTATGTTGG 3'	
EED1MEKR	5'CTCGTTACACGCTTCAAGCTCGCGACC 3'	
CaSymRKENF1	5'TCCGCGGCCGCCATGATGGAGCTACCAGTTATTTG 3'	Cloning of Chickpea SymRK
CaSymRKENF2	5'TCCGCGGCCGCCGAGAGGTACAAAACTTTGATAGGTG 3' (for kinase domain)	
CaSymRKENR	5'GTCGGCGCGCCCTCTCGGTAGTGGGTGTGATAAG 3'	
ENT506448F	5′TCCGCGGCCGCCATGGAACTATCAGATTTGCAAAAC 3′	Cloning of chickpea class-I TCPs in pENTR at *Not*I and *Asc*I restriction sites
ENT506648R	5′GTCGGCGCGCCCTTGAGAGGTATCAGGAGCATC 3′	
ENT492981F	5′TCCGCGGCCGCCATGTCTAACTGCAAGGAAACAAC 3′	
ENT493313R	5′GTCGGCGCGCCCACGATGCTCATCGTCGTCCCGG 3′	
ENT492963F	5′TCCGCGGCCGCCATGGATCCCAAGAGCTCAAAAC 3′	
ENT492963R	5′GTCGGCGCGCCCCTGCCCTGAACCTTGAGAATC 3′	

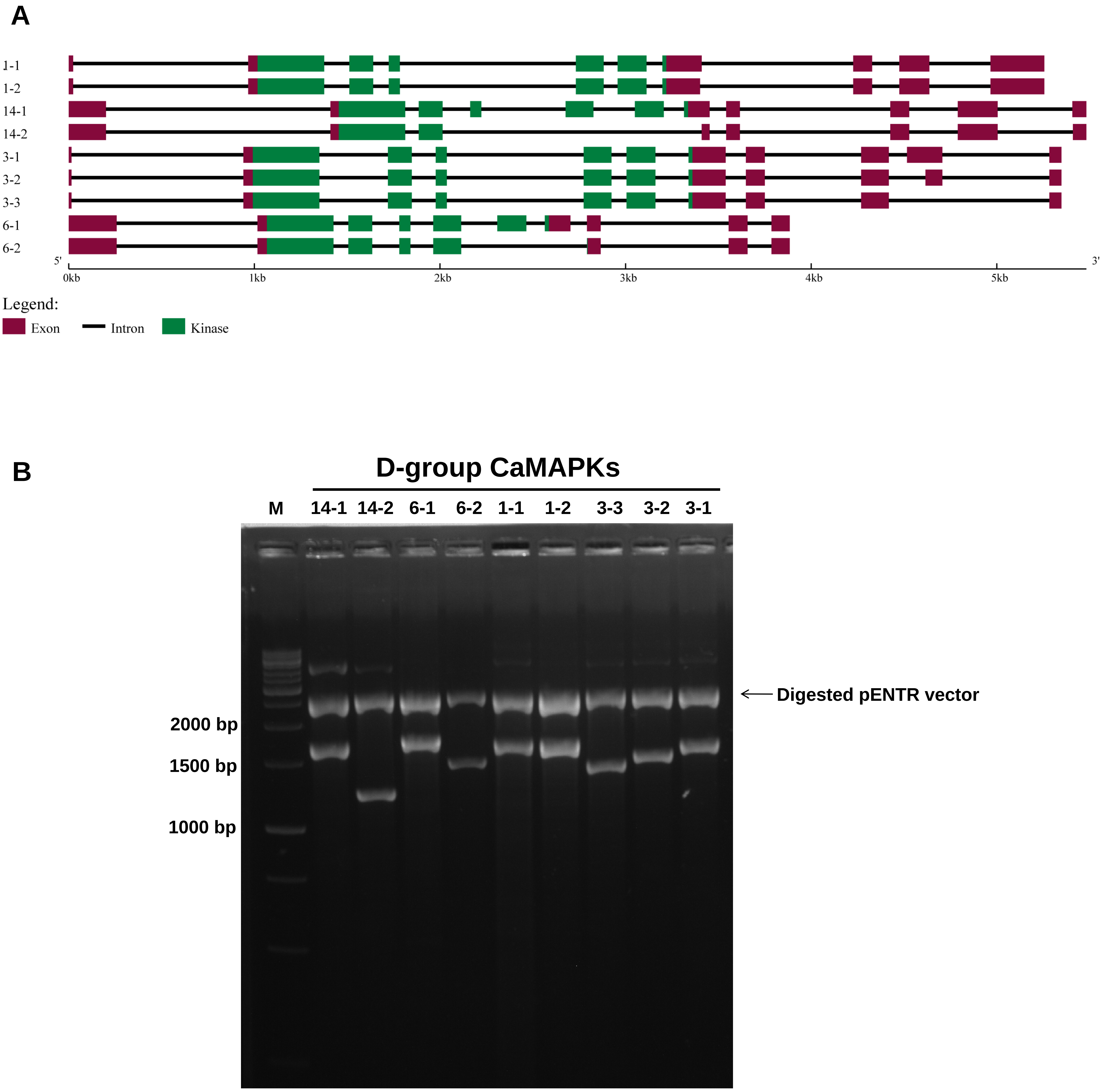

Supplementary Fig. S1. Alternative splicing in D group CaMAPKs.

A. The genomic arrangement of the D group MAPKs splice variants are shown. The genomic and the cDNA sequences were uploaded in the GSDS server to represent the differences in the splice variants. The blocks represent exons and the lines joining them represent introns. The kinase domain is represented in green.

B. Entry clones of the chickpea D group MAPK splice variants in the pENTR vector were digested with *Not*I and *Asc*I showing the difference in insert size.

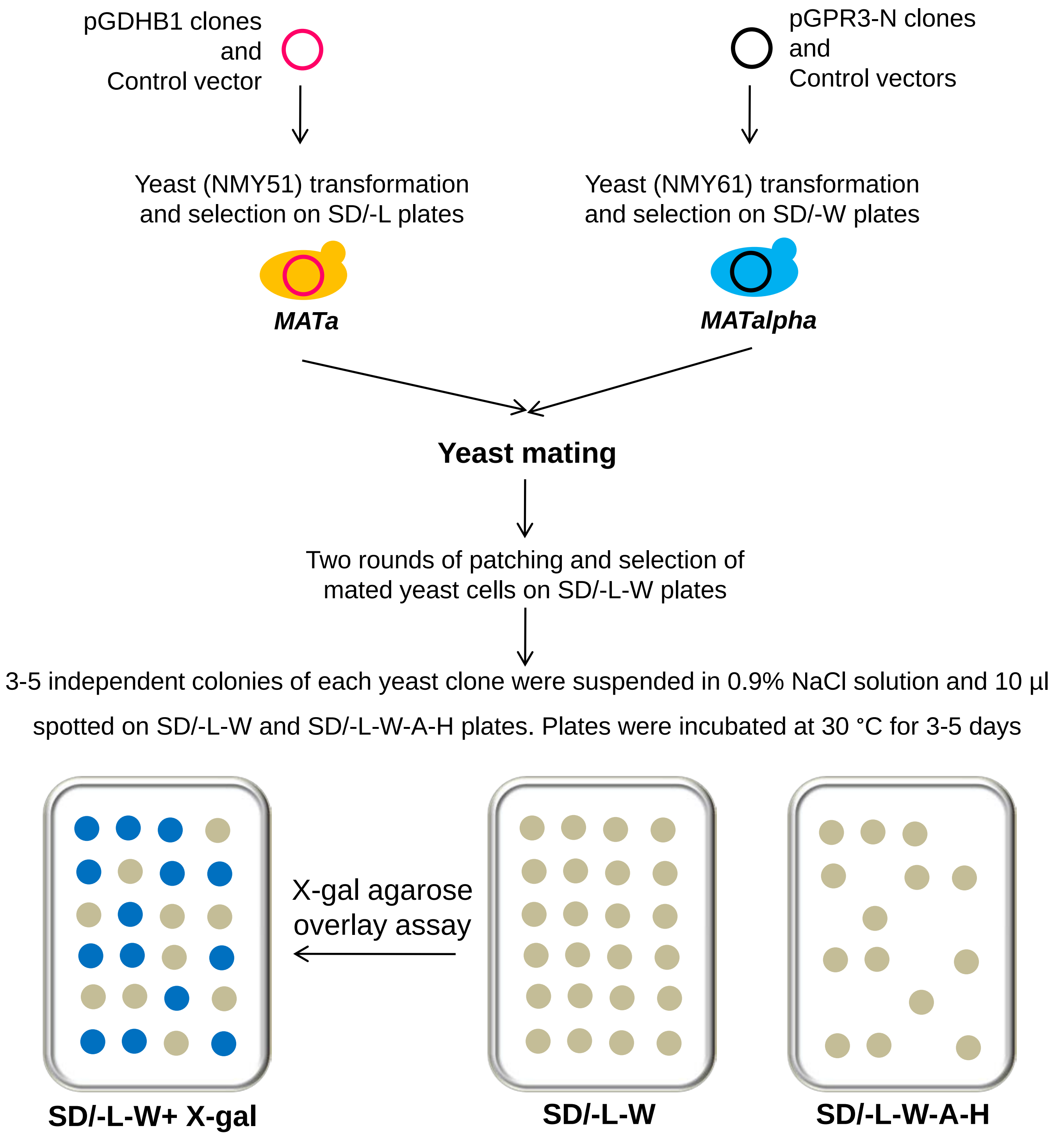

Y2H assay combinations performed (at-least two independent experiments):

7 MKKs + 6 MKK^{EE}s + 20 MAPKs + 3 TCPs in pGDHB1 with pAI-ALg5, pDL2-Alg5 and pPR3-N = **108**

Control pDHB1 with 7 MKKs + 6 MKK^{EE}s + 21 MAPKs + 3 TCPs in pGPR3-N = **37**

7 MKKs + 6 MKK^{EE}s + 3 TCPs in pGDHB1 with 21 MAPKs in pGPR3-N = **336**

20 MAPKs in pGDHB1 with 7 MKKs + 6 MKK^{EE}s + 3 TCPs = **320**

7MKKs in pGDHB1 with 7 MKKs in pGPR3-N = **49**

Supplementary Fig. S2. Representation of comprehensive strategy used for Y2H analysis.

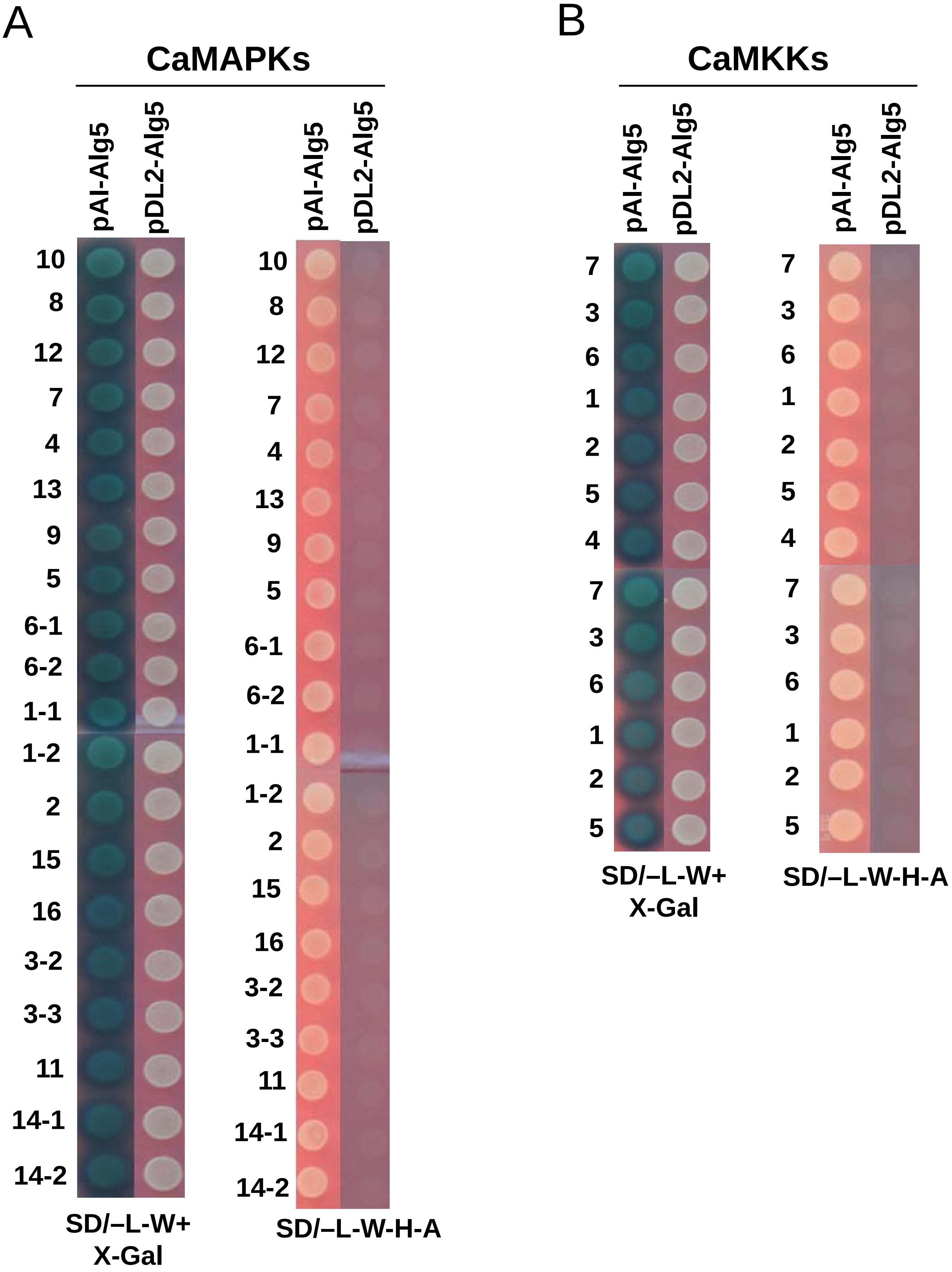

Supplementary Fig. S3. Expression check of MAPK and MKK bait clones

MAPKs (A) and MKKs (B) fused with C-terminal Ubiquitin (Cub) were transformed in NMY51. The bait expression positive control containing the constitutively active form of N-terminal ubiquitin (NubI having isoleucine at position 13) in the vector pAI-Alg5, and the negative control containing the mutated form of N-terminal Ubiquitin (NubG) in the vector pDL2-Alg5 were transformed in the yeast strain NMY61. Mating was performed followed by spotting on SD/–L-W and SD/–L-W-H-A plates. X-gal overlay assay was performed for checking the activation of the *LacZ* reporter gene.

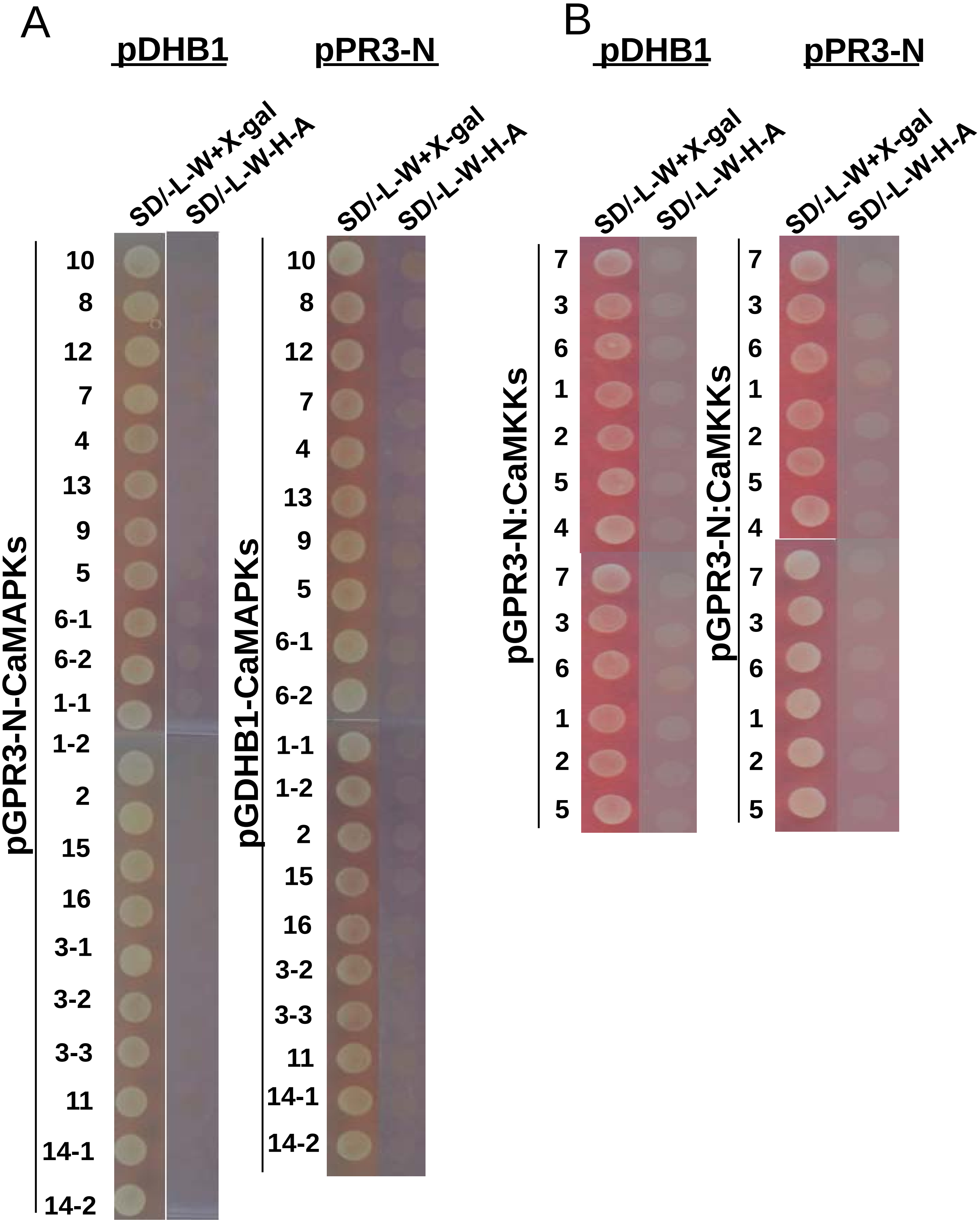

Supplementary Fig. S4. Autoactivation check for MKKs and MAPKs clones

MAPKs (A) and MKKs (B) cloned in pGPR3-N and pGDHB1 were transformed in NMY61 and NMY51, respectively. These yeast cells were mated with NMY51 and NMY61 containing control vectors pDHB1 and pPR3-N, respectively. After mating, the cells were washed and spotted on SD/–L-W and SD/–L-W-H-A plates. X-gal overlay assay was performed for checking the activation of the *LacZ* reporter gene. No yeast growth on selective plates shows CaMKK and CaMAPK clones are suitable for interaction studies.

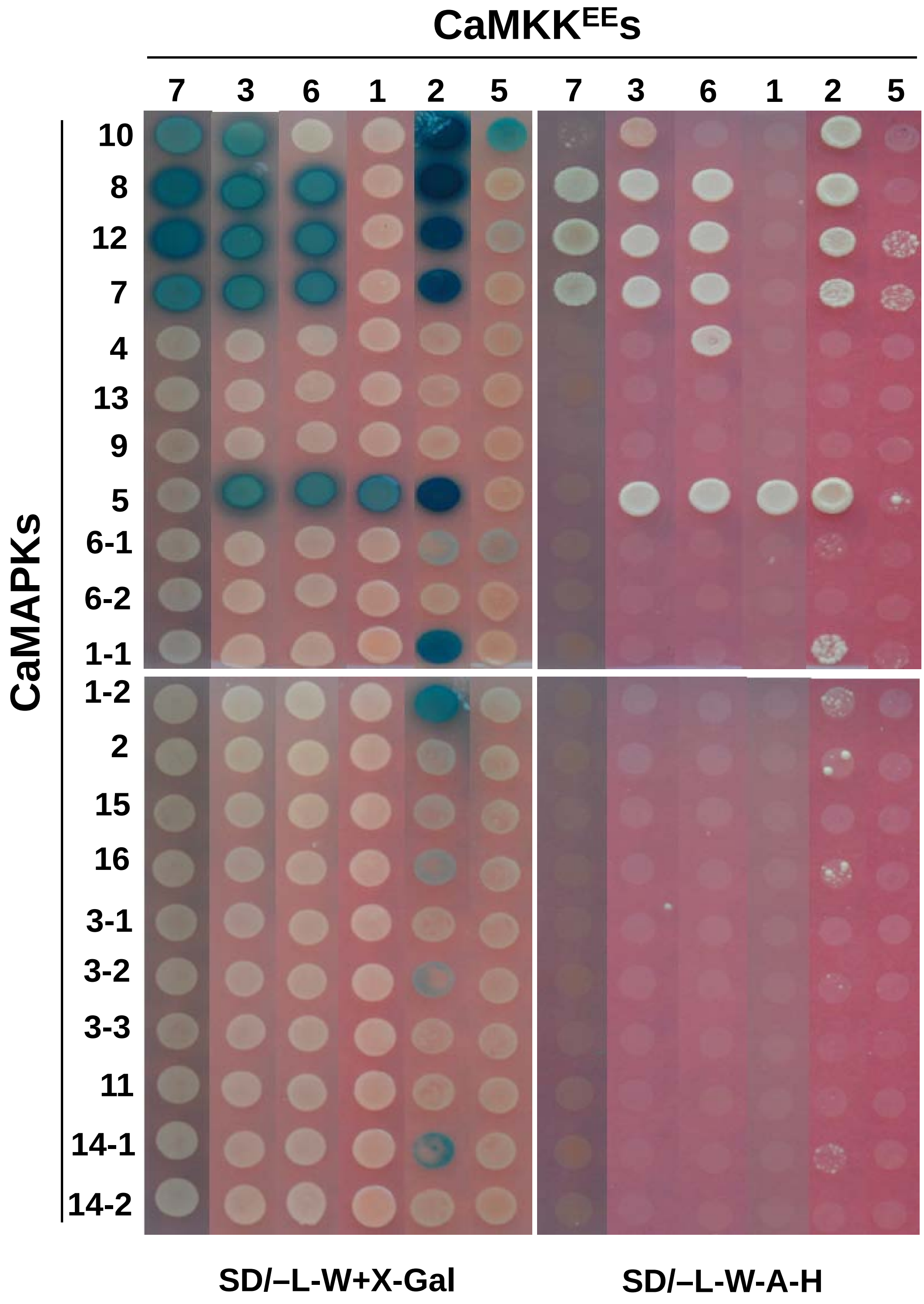

Supplementary Fig. S5. Y2H assay for interaction analysis of MKKs and MAPKs in chickpea. CaMKK^{EE}s and CaMAPKs from chickpea PUSA 362 variety were cloned in pGDHB1 and pGPR3-N vectors, respectively, for an exhaustive interaction study. The plasmids were transformed in separate mating compatible yeast strains. After mating, the cells were washed and spotted on SD/–L-W and SD/–L-W-H-A plates. X-gal overlay assay was performed for checking the activation of the *LacZ* reporter gene. Strong protein-protein interactions showed growth on SD/-L-W-H-A media and blue colour after X-gal overlay assay. Weak interactions showed less growth on SD/-L-W-H-A media and light blue colour.

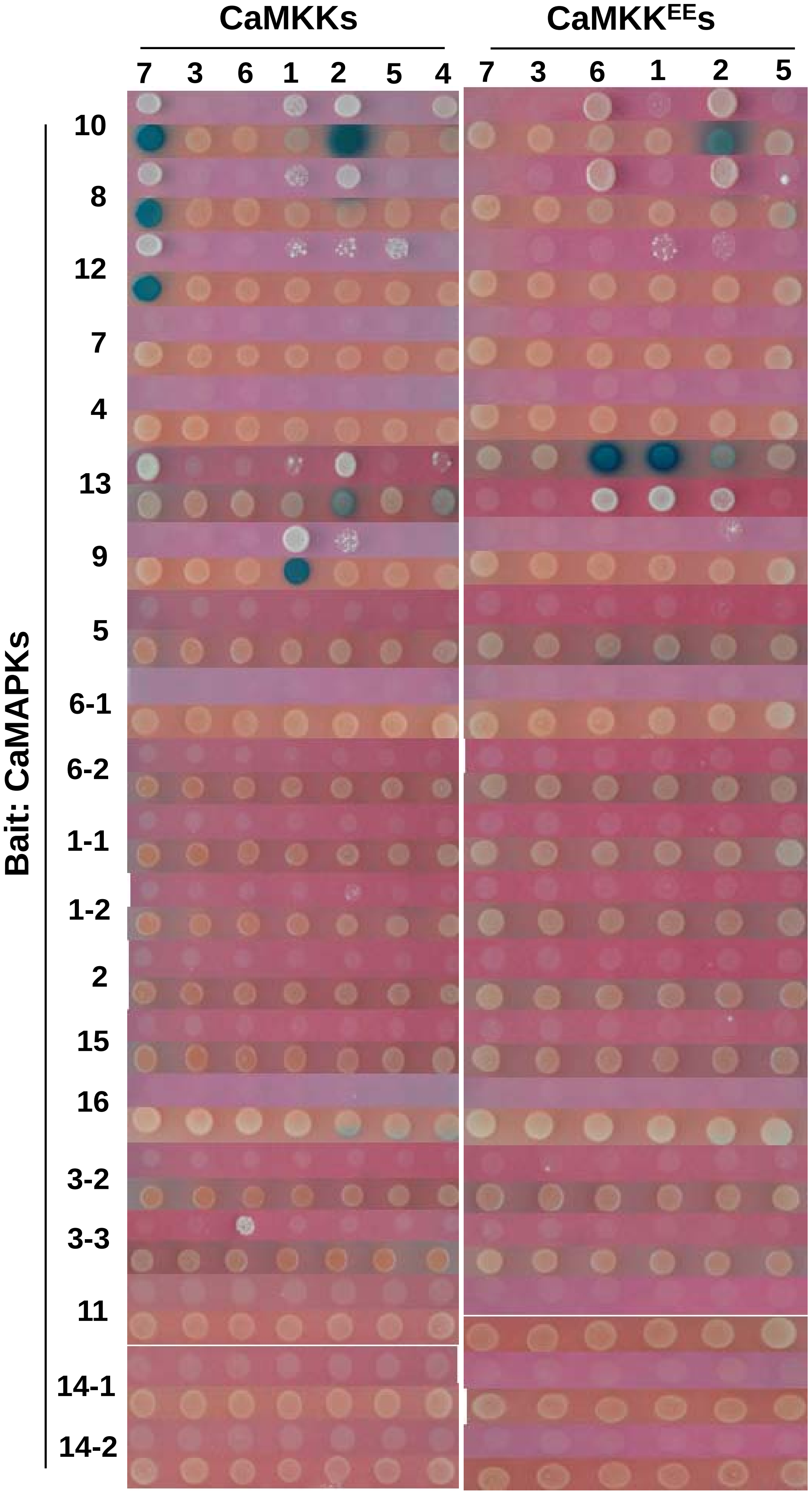

Supplementary Fig. S6. Reciprocal Y2H interaction check for chickpea MKK-MAPK

CaMKKs and CaMAPKs were fused with N-terminal and C-terminal ubiquitin in the Y2H vectors pGPR3-N and pGDHB1, respectively. Yeast growth was checked 48 h after spotting.

A

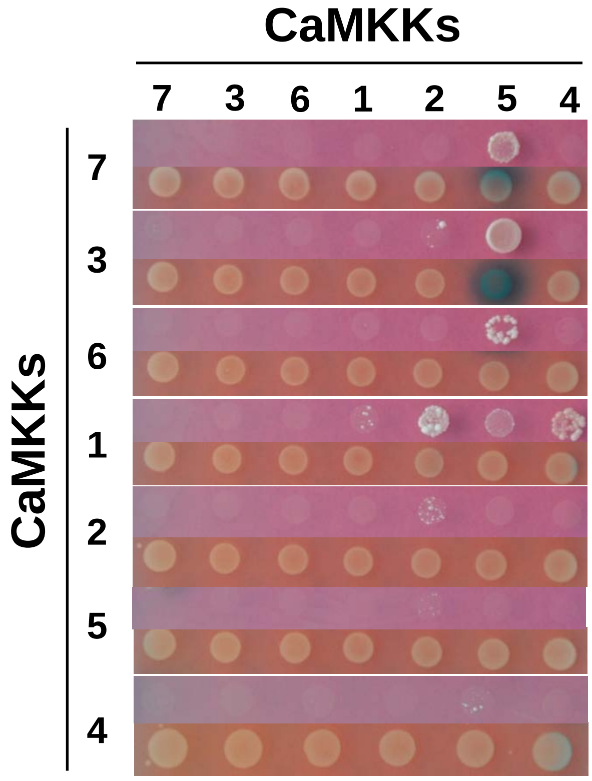

B

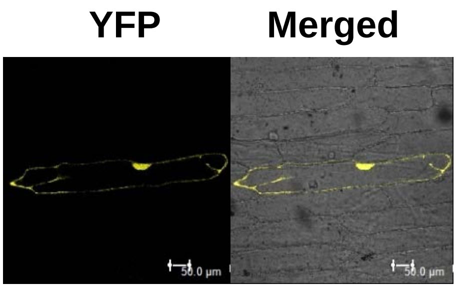

cYFP-MKK5 + nYFP-MKK3

Supplementary Fig. S7. Y2H assay for chickpea MKK-MKK interactions

A) Y2H interaction analysis of chickpea MKKs. Chickpea MKKs were fused with with C-terminal and N-terminal ubiquitin in the split-ubiquitin based Y2H vectors pGDHB1 and pGPR3-N, respectively. pGDHB1 and pGPR3-N clones were transformed in the mating compatible yeast strains NMY51 and NMY61, respectively. Mating was performed and following mating two rounds of spotting was performed on SD/-L-W plates. The yeast cells were then spotted on SD/–L-W and SD/–L-W-H-A plates. X-gal overlay assay was performed.

B) BiFC to confirm the interaction between CaMKK5 and CaMKK3. CaMKK5 was tagged with C-terminal half YFP and CaMKK3 was tagged with N-terminal half YFP. Plasmid DNA of both clones was precipitated on 1µm gold particle and bombarded on onion epidermal cells. YFP fluorescence suggests interaction between CaMKK3 and CaMKK5.

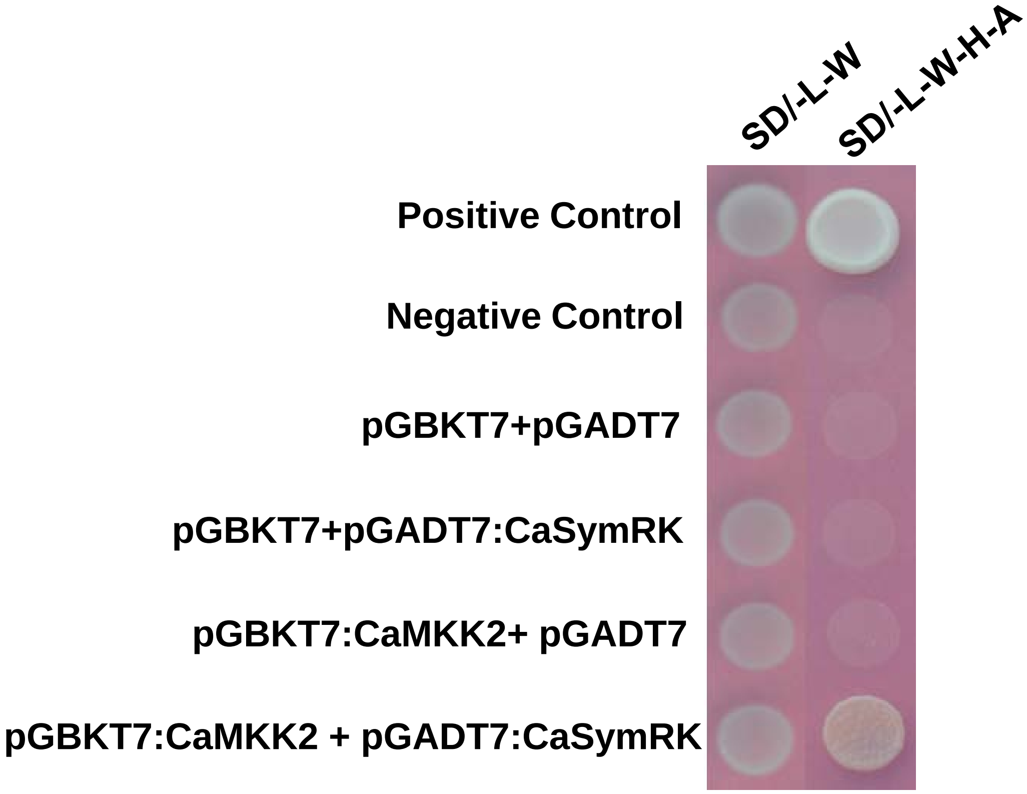

Supplementary Fig. S8. Interaction of CaMKK2 with CaSymRK kinase domain in yeast. CaMKK2 was cloned downstream GAL4 binding and kinase domain of CaSymRK was cloned downstream to the activation domain of GAL4. These clones were co-transformed into the yeast strain Y2H Gold. The transformants were selected on SD/-L-W media. Interaction of CaSymRK kinase domain and CaMMK2 was assessed by ability of the cotransformed yeast cells on SD/–L-W-H-A plates. Results suggest that CaSymRK kinase domain interacts with CaMKK2 in yeast.
